# The reduced kinome of *Ostreococcus tauri*: core eukaryotic signalling components in a tractable model species

**DOI:** 10.1186/1471-2164-15-640

**Published:** 2014-08-02

**Authors:** Matthew M Hindle, Sarah F Martin, Zeenat B Noordally, Gerben van Ooijen, Martin E Barrios-Llerena, T Ian Simpson, Thierry Le Bihan, Andrew J Millar

**Affiliations:** SynthSys and School of Biological Sciences, University of Edinburgh, Edinburgh, EH9 3JD UK; Institute of Structural and Molecular Biology, University of Edinburgh, Edinburgh, EH9 3JR UK; Biomathematics & Statistics Scotland, University of Edinburgh, Edinburgh, EH9 3JZ UK; Institute for Adaptive and Neural Computation, School of Informatics, University of Edinburgh, Edinburgh, EH8 9AB UK

**Keywords:** Conserved eukaryote signalling, Protein kinase phylogeny, *Ostreococcus tauri*, Model kinome, Phosphorylation, TOR signalling, MAPK cascade, Circadian clock

## Abstract

**Background:**

The current knowledge of eukaryote signalling originates from phenotypically diverse organisms. There is a pressing need to identify conserved signalling components among eukaryotes, which will lead to the transfer of knowledge across kingdoms. Two useful properties of a eukaryote model for signalling are (1) reduced signalling complexity, and (2) conservation of signalling components. The alga *Ostreococcus tauri* is described as the smallest free-living eukaryote. With less than 8,000 genes, it represents a highly constrained genomic palette.

**Results:**

Our survey revealed 133 protein kinases and 34 protein phosphatases (1.7% and 0.4% of the proteome). We conducted phosphoproteomic experiments and constructed domain structures and phylogenies for the catalytic protein-kinases. For each of the major kinases families we review the completeness and divergence of *O. tauri* representatives in comparison to the well-studied kinomes of the laboratory models *Arabidopsis thaliana* and *Saccharomyces cerevisiae,* and of *Homo sapiens*. Many kinase clades in *O. tauri* were reduced to a single member, in preference to the loss of family diversity, whereas TKL and ABC1 clades were expanded. We also identified kinases that have been lost in *A. thaliana* but retained in *O. tauri*. For three, contrasting eukaryotic pathways – TOR, MAPK, and the circadian clock – we established the subset of conserved components and demonstrate conserved sites of substrate phosphorylation and kinase motifs.

**Conclusions:**

We conclude that *O. tauri* satisfies our two central requirements. Several of its kinases are more closely related to *H. sapiens* orthologs than *S. cerevisiae* is to *H. sapiens.* The greatly reduced kinome of *O. tauri* is therefore a suitable model for signalling in free-living eukaryotes.

**Electronic supplementary material:**

The online version of this article (doi:10.1186/1471-2164-15-640) contains supplementary material, which is available to authorized users.

## Background

Protein kinases are a major component of the complex signalling networks that coordinate all fundamental cellular processes, including transcription, cell cycle and metabolism. Protein kinases and phosphatases elicit reversible phosphorylation, which enable the rapid cellular responses that are crucial for survival in a continually changing environment. Protein kinases activate and deactivate proteins by addition of the gamma-phosphate from ATP to serine (S), threonine (T), tyrosine (Y), aspartate (D) or histidine (H) amino acid residues [1]. Cascades of consecutive kinase-mediated phosphorylation events constitute the backbone of signalling pathways [2]. The complexity of the signalling networks scales with size. Part of this complexity is constrained by the number of genes encoding protein kinases, also known as the kinome. The number of encoded protein kinases in free-living eukaryotes ranges from as little as 126 kinases in *Saccharomyces cerevisiae* [3] to ~1000 in *Arabidopsis thaliana* [4]. Between these extremes, surveyed organisms include *Dictyostelium discoideum* with 285 kinases [5], the fruit fly *Drosophila melanogaster* with 251, and *Homo sapiens* with 518 kinases [6]. Minimal kinomes are present in parasites that are not obviously representative of other tractable species. The kinome of the parasitic fungus *Encephalitozoon cuniculi* [7] has only 32 kinases and lacks sequences that are ubiquitous in the kinomes of free-living eukaryotes, including the STE family, TOR and AMPK. *E. cuniculi* kinases are also highly divergent within fungi: 9 are reported to have no clear orthologs. The protozoan *Giardia lamblia* can be grown in pure culture and has a small genome of only 6,500 ORFs with a core of only 80 kinases, of which 14 have no clear orthologs and 5 are *Giardia*-specific [8]. The remainder of the kinome is composed of a large expansion of 198 Nek kinases, 139 of which are likely to be catalytically inactive. *G. lamblia* kinase domains were also found to have a mean sequence identity of only 40% with *H. sapiens*, lower than plant and fungal kinases (49-50%).

It was originally thought that S/T and Y kinases were unique to eukaryotes, and that bacteria and archaea operated a parallel system of H and D phosphorylation. However it is now known that S/T and Y phosphorylation is also important in both bacteria [9] and archaea [10]. While many eukaryote-like kinases (ELK) in bacteria share only remote sequence similarity with eukaryotic protein kinases (ePK) they share strong structural similarities [11, 12]. The Rio and Bud32 families of kinases are common to both eukaryote and archaea [11]. Conversely, the Histidine kinases (HK) are also found in eukaryotes, where their roles include osmoregulation in several species [13] and ethylene hormone signalling in *A. thaliana* [14].

A well conserved 250 – 300 amino acid catalytic domain, known as the ePK domain [15], is present in most protein kinases and mediates protein phosphorylation. A small subset of kinases do not possess the ePK domain and are regarded as atypical protein kinases (aPK) [16]. As ePKs are structurally related, a common evolutionary ancestry, distinct from aPKs has been proposed [12]. Members of the protein kinase ePK family [1] are divided into the following major groups: AGC (named after protein kinases A, G and C), TK (Tyrosine Kinases), TKL (Tyrosine Kinase-Like kinases), CaMK (Calcium/Calmodulin-dependent Kinases), CMGC (containing Cyclin-Dependent Kinases (CDK); Mitogen-Activated Protein Kinases (MAPK); Glycogen Synthase Kinase 3 (GSK3) and Cyclin-Dependent Kinase-Like (CKL)), CK1 (Casein Kinase 1), CK2 (Casein Kinase 2), STE (containing homologs of the yeast Sterile kinases), and AUR (Aurora Kinases). The TK family, particularly transmembrane receptor kinases, account for the majority of receptor kinases in humans and serve as cell-surface receptors for growth factors that trigger cell growth, proliferation and differentiation [6]. Non metazoan-eukaryotes, including the green lineage, do not possess genuine TKs [6]. Instead, Y phosphorylation is substituted by dual-specificity kinases that phosphorylate S/T as well as Y [17, 18].

In this study we survey the kinase components of *O. tauri* and assess its suitability as a model organism for eukaryotic signalling, based on two criteria: (1) reduced signalling complexity and (2) conservation of signalling components. *O. tauri* is a promising candidate as it is the smallest free-living eukaryote [19], with a 12.6 Mb genome, encoding 7,989 proteins with minimal genome duplication [20]. This reduced genome might impose simplified signalling. *O. tauri* is part of the Chlorophyta clade within the Plantae supergroup [21], and is taxonomically positioned at the base of the green-plant lineage. Given its size and taxonomic position, it is a promising candidate for generating hypotheses that can be transferred to more complex eukaryotes. *O. tauri* has a streamlined cell structure comprising a single nucleus, mitochondrion, Golgi body and chloroplast [22]. It possesses several benefits as an experimental model, cells can be readily and rapidly cultured in controlled laboratory conditions, where they undergo simple binary cell-division which can be synchronised by light/dark cycles. It has already been used as a model for the eukaryotic cell-cycle, helping to unify current understanding of cell-cycle regulation across eukaryotes [23]. The lack of a cellulose plant cell wall facilitates transformation [24, 25] as well as organelle enrichment and protein extraction [26, 27]. These genetic and proteomic tools have already been applied to studies of protein turnover [27], nutrient deprivation [26] and the plant circadian clock in experimental [25, 28, 29] and mathematical approaches [30].

We survey the *O. tauri* kinome and examine conservation of protein sequences, through phylogenies of kinase orthologs in *A. thaliana*, *H. sapiens* and *S. cerevisiae* as the most widely studied models of plant, metazoan and fungal kinomes respectively. We then focus on three pathways, 1) TOR signalling in *H. sapiens*, 2) MAPK-mediated GSK3 signalling in *A. thaliana* and 3) the core circadian clock. We evaluate the capacity of *O. tauri* components to support signalling in current models of these exemplar pathways. Building on our recent proteomic surveys [26, 31, 32], we examine a large set of phosphorylated peptides detected by mass spectrometry and use these to validate phosphorylation-mediated signalling events in *O. tauri*. In combination with the phylogenetic evidence, we discuss the suitability of *O. tauri* as a model species to study protein kinase signalling.

## Results and discussion

**Figure 1:**
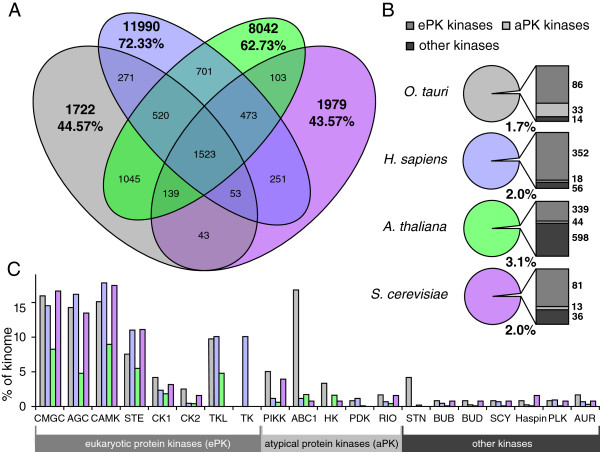
**The proportions of protein-kinase families in** ***O. tauri*** **,** ***A. thaliana*** **,** ***S. cerevisiae*** **, and** ***H. sapiens*** **. (A)** A Venn diagram summarising overall genome similarity in the four species, measured by the inter-species overlap of ortholog-groups predicted by OrthoMCL [33]. Percentages indicate the proportion of the proteome annotated by ortholog-groups that are unique to the organism within this comparison. **(B)** The proportion of each genome that encodes catalytic protein kinases; the adjacent bar charts show the proportion of super-families within this subset. **(C)** The proportions of the genome occupied by each protein kinase family. The families included are those present in *O. tauri*, in addition to TK. “Other kinases”, which are not included in this chart – because they are not present across the eukaryotes studied – amount to 2.5% in *O. tauri*, 59.9% in *A. thaliana*, 9.9% in *H. sapiens*, and 27% in *S. cerevisiae*. The large number of “Other kinases” in *A. thaliana* is due to the many RLKs [34], which are absent in *O. tauri*.

The overall proteomic similarity among *O. tauri*, *A. thaliana*, *S. cerevisiae*, and *H. sapiens* was revealed by a survey of shared ortholog-groups (Figure 1A) identified by OrthoMCL [33]. This approach allows a comparison of shared sequences, despite the widely differing number of protein-family members in their proteomes. The proportion of ortholog-groups that were unique to *O. tauri* and *S. cerevisiae* was very similar, at 45% and 44% of their respective proteomes. *O. tauri* shares 10.6% of the 12,546 ortholog-groups present in *A. thaliana.* The *O. tauri* genome contains a comparable number of *H. sapiens* protein families (2,367) to the existing model species *S. cerevisiae* (2,300).

We compared the number of protein kinases for each family in *O. tauri* with other model organisms, using an existing, high-level classification derived from 22 eukaryotic kinomes [35]. This data-mining approach was augmented by experimental identification of 5,563 phosphorylated *O. tauri* peptides from 107 liquid-chromatography-coupled mass spectrometry (LC-MS) experiments. These correspond to 3,994 uniquely identified phosphorylations of 2,214 peptide sequences of 1,252 proteins (Additional file 1: Table S1), including several conserved protein kinases, discussed below. In the process of identifying and categorising kinases in *O. tauri*, we identified a novel gene locus, corrected 9 existing gene models, and patched sequencing gaps in 25 gene loci with sequence information from *Ostreococcus lucimarinus* data to generate a more complete database for peptide identification. Protein domain diagrams are attached as Additional file 2: Figure S1 while the new and patched gene models and sequences are detailed in Additional file 3: Figure S2. Evidence of phosphorylation motifs conserved between species is presented in Additional file 4: Figure S3.

### *O. tauri*protein kinase and phosphatase survey

A survey of the 7,989 gene models [20] currently annotated in the *O. tauri* genome revealed 133 genes encoding catalytic protein-kinases and 32 protein phosphatases, respectively amounting to 1.7% and 0.4% of the known *O. tauri* loci (Figure 1B, Additional file 5: Table S2). The *O. tauri* kinome occupies a similar proportion of the genome to that found in *S. cerevisiae* (2%, 130 kinases) [3] and *H. sapiens* (2%, 426 kinases) [36] and is proportionally smaller than the *A. thaliana* kinome (3.1%, 981 kinases) [37], with which it shares the greatest sequence similarity of components (Additional file 6: Figure S4).

Phosphatases, in contrast, do not scale with the size of the genome. The Human Phosphatase Portal (HuPho) [38] reports 135 protein phosphatases of which 107 are Protein Tyrosine Phosphatases (PTPs). The remaining 28 S/T phosphatases consist of two families, Metal Dependent Protein Phosphatases (PPMs or PP2Cs) and Phosphoserine Protein Phosphatases (PPPs). *A. thaliana* contains 131 phosphatases of which 10 are PTPs and the remaining S/T phosphatases contain 38 PPPs and 83 PPMs [37]. *S. cerevisiae* contains 25 protein phosphatases [3], which are composed of 6 PTPs, 12 PPPs and 7 PPMs. The *O. tauri* genome contains 32 protein phosphatases, which are composed of 8 PTPs, 10 PPPs and 14 PPMs. The higher proportions of S/T phosphatases to PTPs in *O. tauri* resemble the proportions found in higher *A. thaliana* more than *S. cerevisiae* and *H. sapiens*. The dominance of the PPM family within the S/T phosphatases in *O. tauri* is consistent with *A. thaliana* and *H. sapiens* but is in contrast to *S. cerevisiae*.

A categorisation of kinases into families by sequence similarity and phylogenetic analysis with the *A. thaliana*, *S. cerevisiae*, and *H. sapiens* kinomes confirmed the presence in *O. tauri* of all major ePK families (TKL, CaMK, CMGC, AGC, STE and CK1) present in the green lineage (Figure 1B). We also observed six small, conserved families of ePK-related protein kinases, which are classified as other-ePKs [6] and five families of aPKs. No Receptor-Like Kinases (RLKs) were found in *O. tauri* [39]. The main ePK families account for a large proportion of the kinome in all the eukaryotes. *O. tauri* contains 13 TKL-like kinases, which is consistent with a large expansion of this family in the green lineage [40]. In contrast the TKL family is absent in *S. cerevisiae* and many other fungal genomes [41]. For such a small kinome, *O. tauri* contains a surprising abundance of 20 ABC1-like kinases, which have few functionally-characterised orthologs in other species [42–45]. Recent experimental technologies for targeted gene knock-out in *O. tauri* will therefore greatly assist in the elucidation of their function [46]. Within ePK subfamilies, not all branches are equally conserved, as is evident in the following phylogenetic analyses (Additional file 7: Figures S5, Additional file 8: Figure S6 and Additional file 5: Table S2).

### The TOR pathway: PIKK, CMGC and AGC kinase families

Target of rapamycin (TOR) mediated signalling is vital to the regulation of growth and the key components exist throughout eukaryotes [47]. Here, we describe the phylogenetic relationships within the kinase families that participate in the TOR signalling pathway [48], aPK PI3K-related kinases (PIKK), and the ePK CMGC and AGC kinases.

### PI3K-related kinases (PIKK): TOR, ATR, ATM, TRRAP and DNA-PK

A conserved family of cell-cycle control proteins, phosphatidyl-inositol-3-kinases (PI3Ks) are a class of kinases originally named after their ability to phosphorylate the 3′-hydroxyl group of phosphatidylinositols. The PI3Ks that also act as S/T protein kinases are called PI3K-related kinases (PIKK). Six PIKKs are present in eukaryotic genomes*.* Several of these couple the DNA damage sensing and repair pathway with the control of cell-cycle checkpoints, thereby maintaining the genetic integrity of the genome [49].

**Figure 2:**
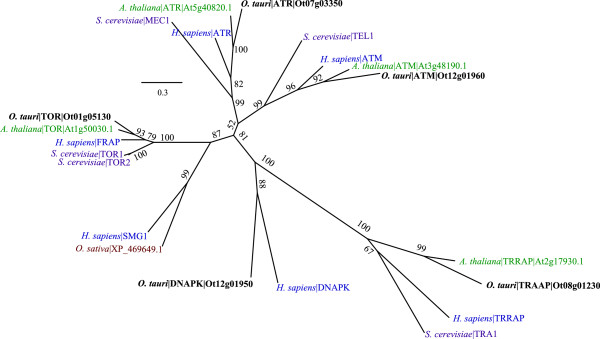
**Phylogeny of the PIKK family.** Sequences from *O. tauri* (bold), *H. sapiens* (blue), *S. cerevisiae* (magenta), *O. sativa* (brown) and *A. thaliana* (green) show the grouping of five *O. tauri* PIKKs into conserved clades. Confidence scores labelled on the edges are bootstrap values. The distance scale is in raw score values from RaXML. Accessions for *O. tauri* sequences refer to the BEG gene models except where we have altered a gene model (Additional file 3: Figure S2). Sequences for *A. thaliana* accessions are from TAIR. Protein identification for PIKK proteins in *A. thaliana* are from Templeton & Moorhead [50]. *S. cerevisiae* identifiers are standard names from SGD. Accessions for *H. sapiens* sequences are given in Additional file 9: Table S3.

The phylogeny of PIKKs (Figure 2) reveal that *O. tauri* contains four of the five PIKK family proteins that are present in plant genomes [50], namely TOR, Rad3-related (ATR), Ataxia-Telangiectasia Mutated (ATM), transformation/transcription domain-associated (TRRAP) protein kinases. The fifth protein, SMG1, is absent in both *O. tauri* and *A. thaliana*, but is represented in *Oryza sativa* (rice). SMG1 has also been identified in 19 other members of the green lineage, and knockouts in *Physcomitrella patens* confirm a conserved role in the nonsense-mediated RNA decay pathway [51]. An additional ATR-like gene fragment (Ot02g03510) is also present in *O. tauri* but was omitted from the phylogeny analysis to prevent gaps in the alignment. The phylogeny consistently groups *O. tauri* sequences into the same clade as *A. thaliana*, distinct from the *S. cerevisiae* and *H. sapiens* group. The topology of the ATM, ATR, and TOR branches of the phylogenetic tree indicates that the *S. cerevisiae* proteins sequences have diverged considerably from *H. sapiens*. Unlike *A. thaliana* and *S. cerevisiae*, *O. tauri* additionally contains a DNA-dependent protein kinase (DNA-PK, Ot12g01950), which groups with the *H. sapiens* DNA-PK in the PIKK phylogeny with an 88% bootstrap confidence (Figure 2). Within the green lineage, candidate DNA-PK orthologs outside the Chlorophyta could only be identified in *P. patens* (XP_001765725) and *Selaginella moellendorffii* (XP_002965996), suggesting that DNA-PK has been lost in higher plants. DNA-PK has a well-defined role in the Non-homologous DNA end-joining (NHEJ) pathway [52], and has recently been recognised in mammals as an important component in the stress-induced phosphorylation of Replication Protein A (RPA) [53]. RPA in turn forms a heterotrimeric complex, which interacts with recombination components to repair DNA double-strand breaks. Unlike DNA-PK, RPA is conserved across eukaryotes, and phosphorylation sites on RPA have been found to be conserved in yeast, metazoa and higher-plants [54]. DNA-PK was also recently shown to be involved in innate immunity against viruses [55]. The presence of DNA-PK in the *O. tauri* kinome makes a first case for *O. tauri* as a model system to study protein kinases (balanced by absence of SMG1), in this case in DNA damage control and potentially in innate immunity.

### CMGC cell cycle family: CDK, MAPK, GSK3

**Figure 3:**
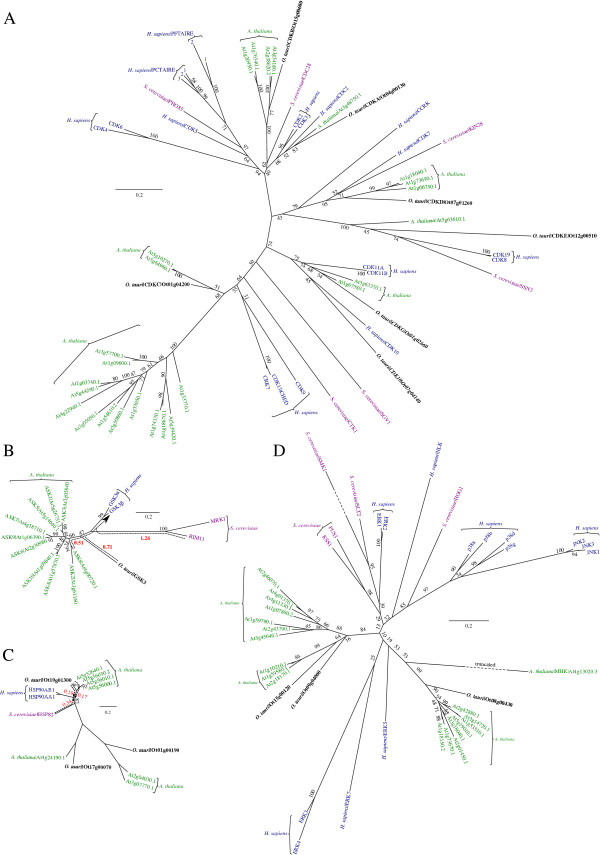
**Phylogeny of CMGC kinases and HSP90. (A)** Phylogeny of CDKs, showing the placement of previously identified cell-cycle kinases in *O. tauri* into the topology of a CMGC phylogeny. **(B)** Phylogeny of GSK3, highlighting the closer proximity of *O. tauri* and *A. thaliana* to the *H. sapiens* sequences (relative to *S. cerevisiae*). **(C)** Phylogeny of HSP90 chaperone, showing a similar topology to GSK3. **(D)** Phylogeny of MAPK, showing specialisation of *A. thaliana* MAPKs into two clades. A general explanation for phylogenies is in Figure 2. In **(C)** all omitted bootstrap values are 100%. Dashed arrows (red) show the distance (sum of branch lengths) from the nearest *A. thaliana*, *S. cerevisiae* and *O. tauri* sequence to the point at which *H. sapiens* sequences diverge, in **B**-**D**.

Among the most conserved of the ePKs are the cell cycle regulating CMGC kinases, which are named after their constituent subfamilies: CDK, MAPK, GSK3 and CLK. We identified 18 CMGC kinases in *O. tauri* (Additional file 5: Table S2) and one CMGC-like gene. Seven are CDKs (Figure 3A) – two of these are CDK10 (Ot07g04140) and the closely related plant-like CDKG (Ot01g02660, Additional file 7: Figure S5A). The five further CDKs are the core cell cycle kinases CDKA (Ot04g00130), CDKB (Ot15g00680), CDKC (Ot01g04200) CDKD (Ot07g01260) and CDKE (Ot12g00510), which are present as single orthologs in *O. tauri* [23, 56], while up to 15 paralogs exist in *A. thaliana*, *S. cerevisiae* and *H. sapiens* (Figure 3A). This makes *O. tauri* a powerful eukaryotic model to study a simplified cell cycle [23].

MAPKs are S/T-specific protein kinases, closely related to CDKs, their growth and stress-response functions – including osmotic shock, oxidative stress and temperature response in plants have been extensively reviewed [57, 58]. We identified 3 plant-like MAPKs in *O. tauri* (Ot08g00430, Ot09g04000 and Ot15g00120, Figure 3D, Additional file 7: Figure S5A), which have 8, 7 and 3 groups of paraologous genes respectively in *A. thaliana*. For MAPK (Ot08g00430) we observed phosphorylation of a conserved Y on the T-X-Y motif of the activation loop (Additional file 6: Figure S4A), which indicates conserved modes of activation. The greatly reduced set of MAPKs in *O. tauri* is a remarkable feature of a highly reduced kinome.

GSK3 is a highly conserved eukaryote CMGC kinase. The chaperone Heat Shock Protein 90 (HSP90) regulates the autophosphorylation of the activating Y in GSK3 [59]. Pharmacological evidence links both HSP90 and GSK3 with circadian timekeeping in *O. tauri* [28]. *O. tauri,* like other algae, has a single copy of GSK3 (Ot04g00510), compared to the ten found in *A. thaliana* [60] (Figure 3B). The *O. tauri* GSK3 kinase domain diverges considerably on its branch between *H. sapiens* and *A. thaliana*. However, the *O. tauri* GSK3 sequence is closer to *H. sapiens* (distance 0.71) than *S. cerevisiae* (1.24). *O. tauri* also contains a single ortholog candidate for HSP90 (Ot10g00440) (Figure 3C), while *A. thaliana* has four HSP90 paralogs [61]. Two closely HSP90-related clades in Figure 3C, acting as outgroups to confirm HSP90 orthology, reveals further *A. thaliana* specialisation of HSPs that is shared in the *O. tauri* genome.

### AGC Kinases: PDK1, S6K and PKG

**Figure 4:**
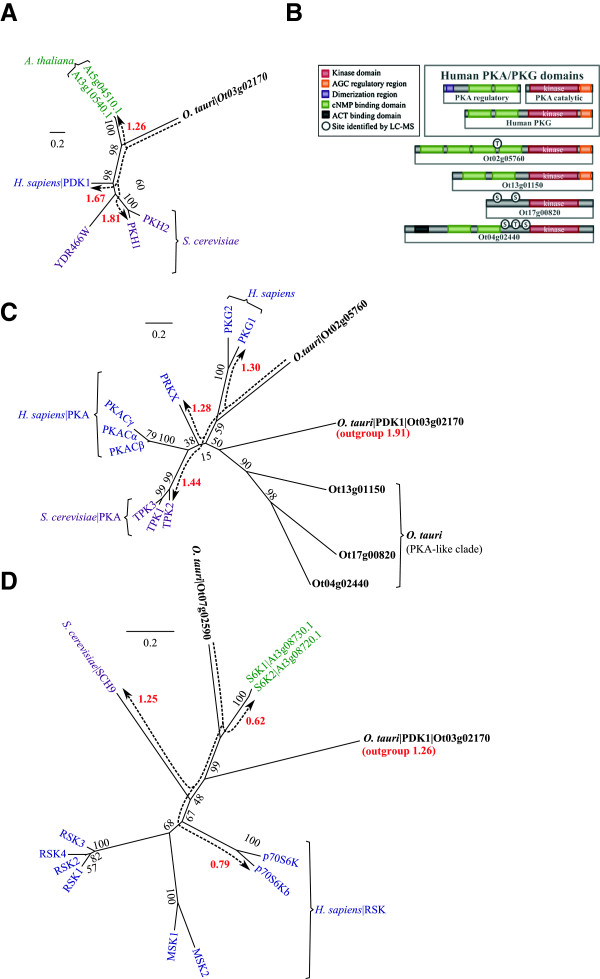
**Phylogeny of AGC kinases. (A)** Phylogeny of the PDK1 master regulator. **(B)** Comparison of domain arrangement for PKA- and PKG-like kinase in *H. sapiens* and *O. tauri*. **(C)** Phylogeny of kinase domains for PKA- and PKG-like proteins and **(D)** S6K phylogeny as compared to the *S. cerevisiae* ortholog (SCH9) and the *H. sapiens* RSKs. A general explanation for phylogenies is in Figure 2. Dashed arrows (red) show distances (sum of branch lengths). The distances shown are from *O. tauri* kinases to their nearest orthologous sequence in each species. *S. cerevisiae* identifiers are standard names from SGD.

Members of the AGC family are cytoplasmic S/T kinases (named after PKA, PKG, and PKC), some of which contain Ca^2+^ sensing domains, regulate glycogen metabolism and ion channel conductance. Phylogeny across diverse eukaryotes has revealed a complex patchwork of conservation that suggests a history of successive contractions and expansions in the AGC kinases [62]. One of the few constants across eukaryote lineages is the Phosphoinositide-Dependent Kinase-1 (PDK1). Within *A. thaliana* only PDK1, Nuclear Dbf2-Related (NDR), Ribosomal S6 Kinase (S6K or RSK) are conserved [63]. Similarly, within *O. tauri* PDK1 (Ot03g02170, Figure 4A), S6K (Ot07g02590, Figure 4D), and NDR (Ot09g00870) are also conserved. Existing work has revealed an expansion of S6K and NDR into large and ubiquitous families across the kinomes of higher plants [40]. PDK1 is present throughout the green lineage but many orthologs, including *O. tauri*, lack a functional lipid-binding domain [62]. PKA- and PKG-like kinases exist in higher plants and other Streptophytes [40] but these have diverged substantially: orthologs from the Chlorophyta such as *O. tauri* have closer sequence similarity to metazoan and fungal sequences than the closest sequences in Streptophytes have to any of these groups. The most PKA- and PKG-like kinase sequence in *O. tauri* is Ot02g05760 (Figure 4B and C). The plant specific light sensor Phototropin1 (PHOT1, Ot16g02900) and Incomplete Root hair Elongation (IRE) (Ot09g04120) kinases are also present. *O. tauri* contains 13 AGC and four AGC-like kinases, of which 5 contain cNMP-binding domains (Additional file 7: Figure S5B).

PDK1 is the most important member of the AGC family in terms of phylogeny as it represents a highly conserved kinase, which has changed little since the divergence of eukaryotic AGCs [62]. PDK1 in *O. tauri* is most similar to the two *A. thaliana* orthologs (1.26). It also groups closer to *H. sapiens* (1.67) than *S. cerevisiae* orthologs (1.81) (Figure 4A). PDK1 is thought to be a basal conserved kinase, which predates the divergence of ePKs [62], and is therefore used to root AGC phylogenies (Figure 4C and D). PDK1 has also been termed the ‘master kinase’ of AGC signal transduction [64] because of its critical role in cellular survival through the activation of Protein Kinase B (PKB, also known as Akt) and S6K in humans [65]. However, out of these two PDK1 targets only S6K (Figure 4D) is conserved in *A. thaliana* and *O. tauri*.

The cAMP-dependent protein kinases (PKAs) and cGMP-dependent protein kinases (PKGs) are part of the same sub-family of kinase domains [36] and have similar domain components, and quaternary structure [66]. PKG is composed of a single protein with cGMP binding and protein-kinase activity. PKA is a heterodimer composed of separate protein-kinase and cAMP binding subunits. The inactive complex disassociates when cAMP binds to the regulatory subunit, which releases the active protein-kinase component [67]. As with many AGC proteins, a conserved C-terminal tail acts as a phosphorylation site for priming the protein-kinase active-site [67]. There are five AGC kinases with cNMP binding domains in *O. tauri*. Two of these (Ot02g05760 and Ot13g01150) contain all three domain components and have kinase domains with the strongest similarity to *H. sapiens* PKAs (Figure 4B and Additional file 2: Figure S1). Ot13g01150, has the closest domain structure to *H. sapiens* PKA/PKG, and appears at the base of a subclade with two other kinases (90% confidence), branching prior to the divergence of PKA and PKG (Figure 4C).

Ot02g05760 is assigned with low confidence (59%) to the base of the PKG branch. However, it diverges near to the root of *H. sapiens* PKA-like kinases, which results in the domain being closer to PRKX (1.28) than to PKG (1.30); PRKX is part of the family of PKA catalytic subunits [68]. This supports a PKA like activity for the domain, rather than the more constrained PKG substrate specificity [69].

### The minimal TOR Pathway in *O. tauri*: An inventory

TOR is highly conserved across eukaryotes and acts as a master regulator for nutrient-responsive growth in yeast, metazoa [70], and plants [71]. S6K1 and S6K2 are targets of the TOR pathway in *A. thaliana* [71], and rapamycin inhibits this pathway, as in other organisms. S6K contains a conserved C-terminal motif that is a target for TOR phosphorylation and PDK1 binding, and this motif is highly conserved in *O. tauri*. In mammals, complexes of mTOR with RAPTOR (TORC1) and RICTOR (TORC2) mediate distinct signalling pathways. The LST8 protein is a common component of both complexes. Equivalents for both mTOR complexes exist in yeast [72, 73]. *O. tauri*, like the rest of the green lineage, only contains components of TORC1. The kinase targets of TORC2 (PKB, PKC) are absent from *O. tauri*, and across the green lineage. In contrast, S6K is a conserved as a target of TORC1 in the green lineage [71], suggesting that the TORC1-containing mTOR complex could be the prototypical pathway for TOR signalling.

**Figure 5:**
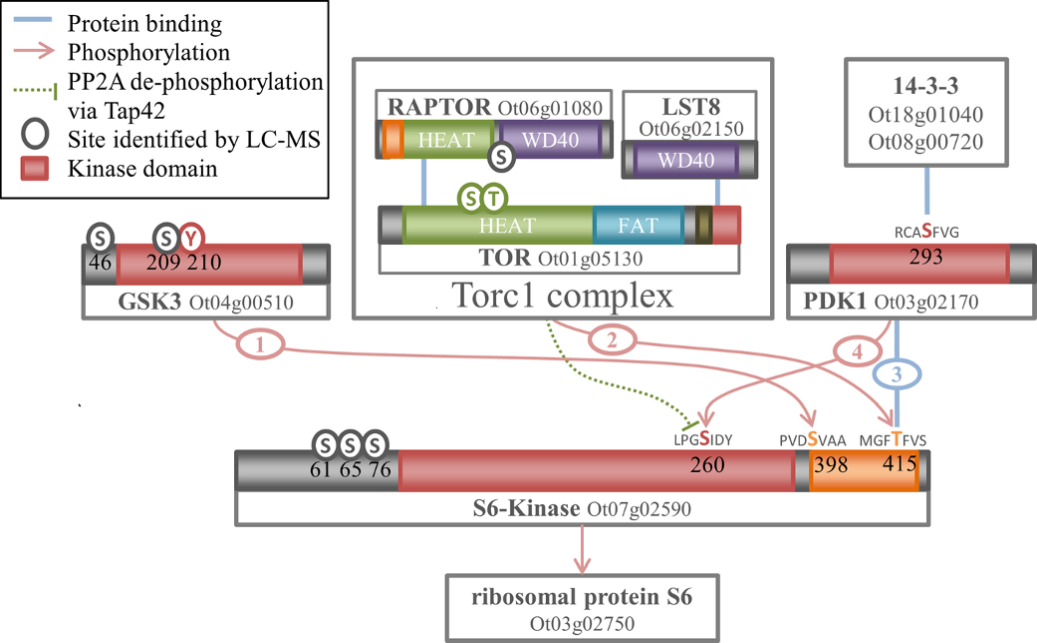
**Schematic of the regulation of TOR signalling and S6K activation in** ***O. tauri*** **.** Numbered connectors indicate the order of the stages of S6K activation, as proposed by Shin *et al.*[48]. (1) GSK3 phosphorylates S398 allowing the (2) phosphorylation of T415 by the TORC1 complex. (3) Phosphorylated T415 is able to bind PDK1, which phosphorylates S260, leading to the activation of S6K. Additionally, TOR mediated inhibition of S6K is shown, which may occur via TAP42, previously unannotated in the *O. tauri* genome. TAP42 in yeast binds PP2A and prevents the latter from dephosphorylating the GSK3 target site of S6K.

Having established the phylogenies for the essential TOR pathway components GSK3, TOR, PDK1 and S6K [48, 74–76] and its essential regulators in *O. tauri*, we now turn to analysing the complex series of phosphorylation events of the TOR pathway. The conserved *O. tauri* components required for GSK3-mediated S6K regulation are shown in Figure 5, overlaid with the mammalian interactions based on conserved phosphorylation motifs and binding sites. It has been suggested that GSK3 may initiate the activation process of S6K [48]. This involves a complex series of phosphorylation events by multiple components. Activated S6K transmits the final TOR pathway signal by phosphorylating the ribosomal protein S6, initiating it to regulate the translation machinery. Upstream, the activation of S6K is proposed to require the concerted action of three phosphorylation events by GSK3, the TORC1 complex and PDK1, in this order.

In mammals, the first of these phosphorylation events is by GSK3 to the equivalent residue of S398 on the S6K turn motif [48]. The GSK3 target S/T-X-X-X-S/T motif at this site is conserved in *S. cerevisiae*, *A. thaliana*, and *O. tauri* (Additional file 4: Figure S3B). This residue is constitutively phosphorylated in mammals, and is a dephosphorylation target of PP2C. The presence of GSK3 is proposed to infer resistance to PP2C-mediated inactivation of S6K by countering dephosphorylation [48]. The phosphorylation of S6K by GSK3 at S398 is a pre-requisite for the subsequent phosphorylation of the C-terminal T415 by TOR. The phosphorylation by TOR in turn enables the binding of active PDK1 [77] to the C-terminal motif. The activation of human PDK1 requires autophosphorylation of a S in the activation loop, which is also a 14-3-3 binding motif [78]. Human 14-3-3 binds to the phosphorylated motif in PDK1 [79]; 14-3-3 also regulates PDK1 in *A. thaliana* [80]. The activation-loop S is conserved in *A. thaliana* and *O. tauri* PDK1 (S210). There are only two 14-3-3 proteins in *O. tauri* (Ot18g01040 and Ot08g00720), providing a limited number of candidates for PDK1 regulation. Active PDK1 binds to the primed TOR motif at the C-terminus of S6K (T415). This allows PDK1 to phosphorylate a T residue in the S6K activation loop [74, 75], which is also conserved in *A. thaliana* and *O. tauri* (S260). Yeast and human TOR phosphorylates TAP42 (or α4 in humans), which affects the formation of a TAP42:PP2A complex [81]. PP2A has been shown to dephosphorylate S6K [82], but it is unclear what role this potential signalling pathway has on S6K regulation in higher eukaryotes [83]. Both TAP42 (Additional file 4: Figure S3) and PP2A (Ot07g01700) are found within *O. tauri* and the green lineage, though the TAP42 ortholog in *O. tauri* was previously unannotated.

We have shown conservation of the AGC kinases in the TORC1 pathway in *O. tauri*. Key phosphorylation motifs and binding sites are also conserved, for all the components of the model proposed by Shin *et al.* [48]. No phosphorylation was detected for the three key residues of S6K in our phosphoproteomic surveys. S6K was present, as phosphorylation at S61, S65, and S76 were detected and similar samples observed the unphosphorylated protein [26]. However, the lack of detected phosphorylation in *O. tauri* cannot be taken as contrary evidence as the quantity of observed phosphorylation in proportion to expected phosphorylation in *O. tauri* is still relatively low. For example, 28 phosphorylations of human S6K are currently known [69], and assuming a similar quantity of modification in *O. tauri* we have observed in the order of 10% of phosphorylations.

### The CaMK family: CPK and SnRK1 (ePK)

**Figure 6:**
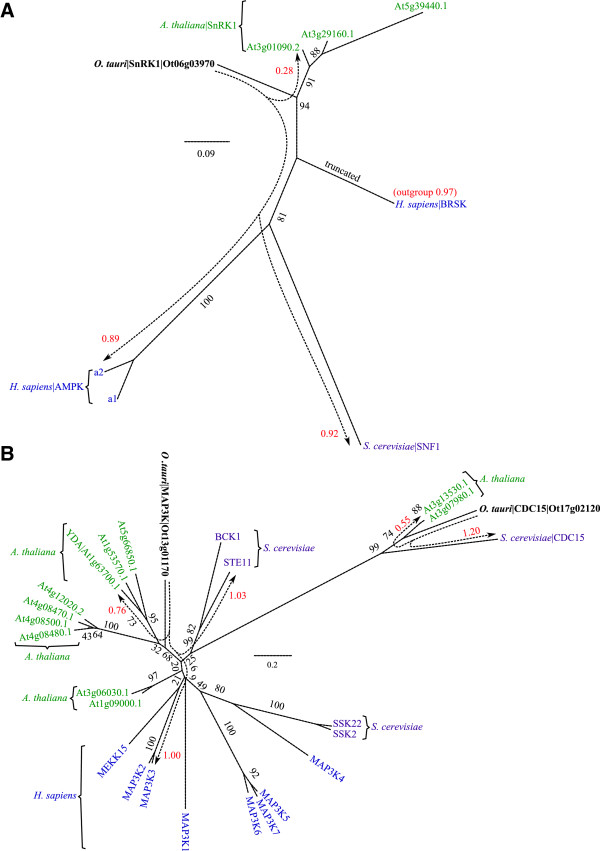
**Phylogeny of SnRK1s and MAP2Ks. (A)** Phylogeny of SnRK1 showing orthology with SNF1 in *S. cerevisiae* and AMPKs in *H. sapiens*. Brain-Specific Kinase (BRSK) acts as an outgroup. The full set of SnRKs are reported in Additional file 7: Figure S5D. **(B)** Phylogeny of MAP3K revealing distinct YDA- and CDC15-like MAP3Ks in *O. tauri*. It shows a strong conservation within the *S. cerevisiae* and plant cell cycle kinase CDC15 (not present in *H. sapiens* or other metazoa). The large numbers of MAP3K proteins in other species are reduced to a single member in *O. tauri*. A general explanation for phylogenies is in Figure 2. Raw branch lengths (red) are annotated to broken-line arrows and show the distances from *O. tauri* kinases to their orthologous sequences in *A. thaliana*, *S. cerevisiae*, and *H. sapiens. S. cerevisiae* identifiers are standard names from SGD.

The CaMK are an important family of S/T-specific kinases with functionally divergent roles, which are represented by five *O. tauri* genes and thirteen CaMK-like genes that are more distantly related (Additional file 5: Table S2). Consistent with previous findings [84], plant CaMKs are highly divergent with yeast and human proteins. Hence, we sub-classified the *O. tauri* CaMKs based on the separate phylogenies of two plant sub-families: Calcium-Dependent Protein Kinases (CDPK; Additional file 7: Figure S5C) and Sucrose-nonfermentation1-Related protein Kinases (SnRK, Figure 6A; Additional file 7: Figure S5D). In plants CaMKs have a large diversity of roles in extracellular signalling and target substrates in pathways such as carbon and nitrogen metabolism, homeostasis, transcription and proteasome regulation [85].

SnRK are an important subfamily of conserved CaMKs, which are related to SNF1 in yeast. SnRK1 kinases are the founding members and are most closely related to SNF1. It is also the only member of the SnRK family which is present across all eukaryotes and core members are involved in energy regulation in the cell [86, 87], with a primary function in glycogen metabolism [88]. The *O. tauri* SnRK1 (Ot06g03970) is most closely related to the *A. thaliana* SnRK1s (Figure 6A; Additional file 7: Figure S5D). In addition to the core SnRKs, *A. thaliana* contains a functionally diversified set of SnRK subfamilies [86], which are absent from *O. tauri*. The human ortholog 5′ AMP-activated Protein Kinase (AMPKa) is equidistant to *O. tauri* and the *S. cerevisiae* ortholog SNF1, indicating *O. tauri* may also be a suitable model for SnRK signalling in humans (Figure 6A).

CDPKs have many roles in biotic and abiotic signalling pathways [85]. Two kinases exist in *O. tauri* (Ot09g03470 and Ot03g03430) with clear CDPK domain architectures and kinase domains. These have recently been classified as group I algal CDPKs [89]. CDPKs are typically a large family, involved in a variety of roles specific to higher plants, including herbivore defence [90] and abscisic acid signalling [91–93]. These specialised functions indicate that kingdom-specific adaptations have driven the sequence diversity of CDPKs. A Phosphoenolpyruvate carboxylase-Related Kinases (PEPKR) Ot01g05370 is also present in *O. tauri* (Additional file 7: Figure S5C). Two other *O. tauri* kinases align more closely to the *S. cerevisiae* RAD53 (Ot15g01210 and Ot07g01980) than CDPKs. Ot15g01210 has previously been classified as a group IV algal CDPK [89]. Recent CDPK phylogenies by Hamel *et al.* [89] have shown that plant CaMKs are likely to have diverged between the emergence of group IV and I, which places Ot15g01210 and Ot07g01980 within two distinct clades for plant CDPKs. The retention of group IV and I clades within the reduced *O. tauri* kinome suggests an important conserved role for these kinase in calcium signalling.

### The MAP2K pathway and STE kinase family

The downstream effects of extracellular signals, which are mediated by kinases such as the CaMKs and AGCs, are the MAPK cascades. These form signalling connections from the cellular environment into the nucleus, in order to affect transcriptional changes [2]. All but the final target of the MAPK cascade are found within the STE family. Here we examine the STE family of kinases with a view to understanding an exemplar MAPK pathway from *A. thaliana*: the GSK3-mediated regulation of stomatal opening through a MAP2K target [94, 95]. As for the previously described TOR pathway, we first assess the relevant kinase orthologs in *O. tauri*.

### STE kinases

The STE kinases contain the MAP4K, MAP3K and MAP2K components of the MAPK cascade [6]. Eight STE family kinases and an additional six STE-like kinases were identified in *O. tauri* (Additional file 7: Figure S5E). These include one MAP2K (Ot04g04050), two MAP3K (Ot13g01170 and Ot17g02120, Figure 6B), and two MAP4K (Ot02g05830 and Ot13g02030) kinase candidates. In contrast, *A. thaliana* has 10, 11 and 7 orthologous genes respectively, again emphasizing the potential of *O. tauri* as an experimental model for gene manipulation in MAPK signalling studies. Within MAP3Ks Ot13g01170 is the only member of the MEKK clade and Ot17g02120 is a CDC15-like protein. The STEs are closely related to the Tyrosine-Kinase like (TKL) family, and contain the plant-RAF kinase, which also act as MAP3Ks [96]. Ot12g01310 is the only confirmed plant-RAF kinase (Additional file 5: Table S2). It contains a Constitutive Triple Response 1 (CTR1) domain (Additional file 2: Figure S1), confirming it as an ortholog of the *CTR1* gene: a potential-MAP3K that in *A. thaliana* is negatively regulated by the ethylene responsive histidine kinase ETR1 [97].

*O. tauri* also contains a single plant-like APG1 kinase (Ot06g01800) with four orthologous proteins in *A. thaliana*. C-terminal phosphorylation of APG1 was observed in *O. tauri*. APG1 kinases in yeast and *A. thaliana* are a target for the negative regulation of autophagy by TOR [98, 99], highlighting another conserved facet of the TOR pathway.

### The minimal MAP2K Pathway in *O. tauri*

The brassinosteroid signalling pathway acts upstream of GSK3 in *A. thaliana*, to initiate GSK3-mediated inhibition of the MAPK pathway, leading to stomatal regulation [95]. The central components of this pathway are found in *O. tauri*; however, neither the upstream brassinosteroid signalling pathway nor the downstream stomatal regulation components are present. Similarly, in human and yeast cells MAPK cascades create complex signalling networks in a diverse array of processes [2, 100], many of which are absent in *O. tauri*. Despite the diversity of processes, these central MAPK components from the CMGC and STE kinase families are among the most conserved protein kinase families in *O. tauri* (Additional file 7: Figure S5).

**Figure 7:**
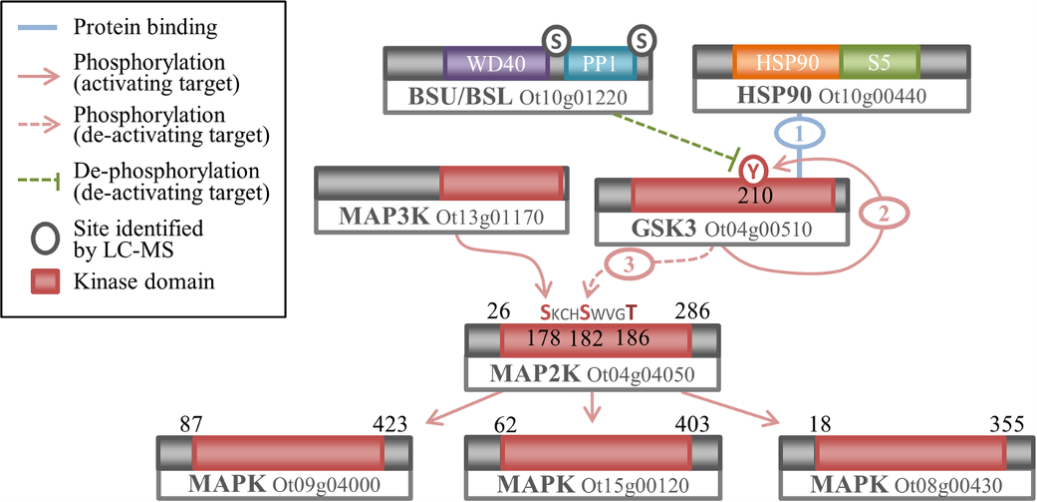
**Schematic of GSK3 mediated signalling through the MAPK cascade in** ***O. tauri*** **.** BSU has been shown to dephosphorylate a Y to inactivate GSK3 in plants [102], which is conserved as Y210 in GSK3. HSP90, which is conserved across eukaryotes has been shown to chaperone the autophosphorylation of the activating Y210 in GSK3 [59]. Conservation of S182 and T186 in the *O. tauri* MAP2K form part of a GSK3 phosphorylation motif, which when phosphorylated inhibit MAP2K activity in *A. thaliana*[94]. The upstream S182 is a conserved residue required for activation by MAP3K.

The residues of MAP2K that are proposed to be part of the GSK3 phosphorylation motif S/T-X-X-X-S/T are conserved in *O. tauri*, *A. thaliana*, *S. cerevisiae* and *H. sapiens.* The conserved residues are S182 and S186 in the *O. tauri* MAP2K (Ot04g04050). The residues S178 and S182 are conserved with those required for activation of MAP2K in human (MEK1) and yeast (STE7) [101]. The motif surrounding S178 appears to be more variable across eukaryotes than the downstream GSK3 motif. The residue corresponding to S178 is phosphorylated by a MAP3K (YODA) in *A. thaliana* [94]. Figure 7 shows the *O. tauri* components associated with the MAP2K pathway, along with a proposed schema based on the current *A. thaliana* model. The BSU family of green-lineage phosphatases, shown to regulate GSK3, are represented by a single member in *O. tauri*. The MAPK pathway, which GSK3 inhibits in *A. thaliana*, also has only a small number of possible components in *O. tauri*.

### Circadian signalling: CK1 and CK2

Circadian rhythms are ≈ 24 h biological cycles, which arose as adaptations to daily changes in the environment. The circadian clock regulates diverse processes across eukaryotes, from the sleep–wake cycle of metazoa to photosynthesis [103]. *O. tauri* is already in use as a clock model for both *in vivo* and *in silico* studies [25, 30, 104]. In particular CK1 and CK2 have been shown to be part of conserved transcriptional/translational feedback loops in eukaryotes that regulate circadian clocks, based on pharmacological and overexpression results [28, 31, 32].

### Casein Kinase 1 family

The CK1 family of kinases are named after the highly conserved CK1 protein. CK1 has a variety of cellular functions, including regulation of membrane trafficking, DNA replication, Wnt signalling, RNA metabolism [105] and cell cycle regulation through tubulin binding [106, 107]. CK1 isoforms have also been shown to affect circadian rhythmicity in metazoa [108, 109], in the fungus *Neurospora crassa* [110] and in *O. tauri* [31, 32].

**Figure 8:**
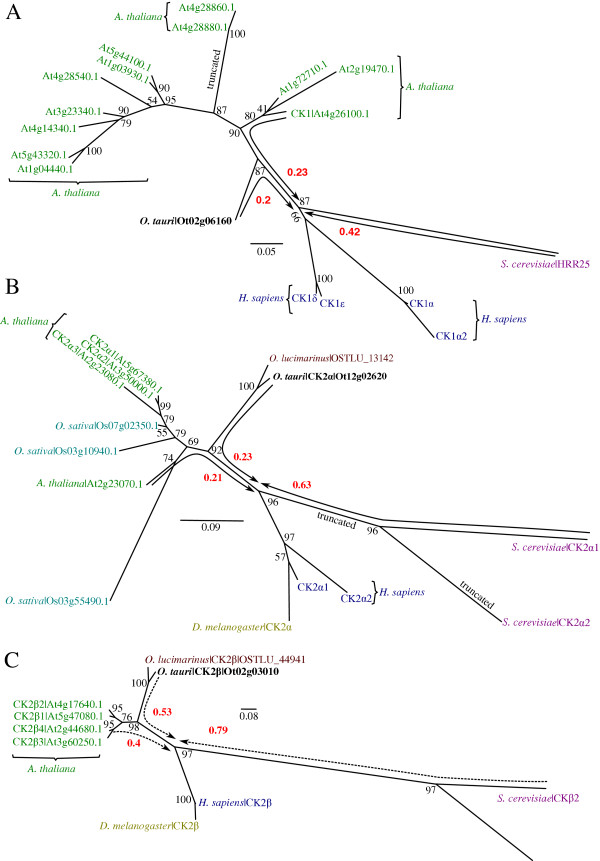
**Phylogenies CK1, CK2α and CK2β. (A)** Phylogeny of CK1, revealing an *O. tauri* ortholog in close proximity *H. sapiens* (relative to *A. thaliana* and *S. cerevisiae*). **(B)** Phylogeny of CK2α catalytic subunit showing the closer proximity of *A. thaliana* CK2α to the *H. sapiens* sequences (relative to *S. cerevisiae*). **(C)** Phylogeny of CK2β regulatory subunit, revealing a similar topology and rate of relative divergence to the CK2α subunit. A general explanation for phylogenies is in Figure 2. Raw branch lengths (red) are annotated to broken-line arrows and show distances from *O. tauri*, *A. thaliana*, and *S. cerevisiae* to the *H. sapiens* and *D. melanogaster* divergence point. For **(B)** the *H. sapiens* CK1γ isoforms are not shown, as they are more divergent than the other sequences included. The CK2α2 branch has been truncated as it was considerably more divergent than CK2α1. *S. cerevisiae* identifiers are standard names from SGD. Accessions for *D. melanogaster* and *H. sapiens* proteins are given in Additional file 9: Table S3.

CK1 typically consists of a large number of paralogs per organism, a selection of which is shown in the phylogeny in Figure 8A (Additional file 7: Figure S5F). The human kinome contains seven isoforms (CK1α-ϵ), of which the four least divergent are shown. The *H. sapiens* CK1δ and CK1ϵ form the first branch after the divergence of *S. cerevisiae*, with a bootstrap confidence of 99%. *A. thaliana* contains many CK1 paralogs, of which CKL1-13 are the most conserved. The closest *A. thaliana* CK1 has a distance of 0.23 to the point at which *H. sapiens* and *S. cerevisiae* diverge from the other sequences (Figure 8A). *S. cerevisiae* encodes three CK1 isoforms, of which the closest (HRR25, with a distance of 0.42) is included in Figure 8A. The *O. tauri* CK1 sequence (Ot02g06160) branches near the midpoint between *A. thaliana* and the *S. cerevisiae* and *H. sapiens* sequences, only a relatively short distance of 0.2 away from the *S. cerevisiae* and *H. sapiens* branch. *O. tauri* contains four further CK1-like protein kinases, of which only one (Ot02g06100) is conserved in *A. thaliana* (Additional file 7: Figure S5E).

Based on taxonomic studies of eukaryotes [21, 111] we generally expect the Chlorophyta branch of algae and plant proteins to be more closely related, with a smaller distance between sequences from metazoa and yeast. However, our data shows that the distance of the *O. tauri* CK1 to the base of the branch that contains *H. sapiens* sequences is less than half the distance of *S. cerevisiae* to this branch. *A. thaliana* CK1 sequences also appear to have diverged considerably. The *O. tauri* CK1 is the closest among these three model organisms to human CK1δ and CK1ϵ. This indicates that *O. tauri* may be an interesting model organism to study CK1.

### Casein Kinase 2 (CK2)

Casein Kinase 2 (CK2) is a highly conserved kinase, found across all eukaryotes. CK2 is centrally important in many signalling pathways and is one of the most ubiquitous kinases in terms of substrate phosphorylation [112]. CK2 is a tetramer composed of a CK2β dimer and two CK2α subunits*. O. tauri* contains a single catalytic CK2α (Ot12g02620) and regulatory CK2β (Ot02g03010) subunit. The topology of the phylogenies for both subunits is very similar, the *O. tauri* CK2 appears to be more similar to the *A. thaliana, D. melanogaster* and *H. sapiens* than *S. cerevisiae* sequences. *S. cerevisiae* CK2 subunits have diverged considerably, similar to CK1, indicating that *O. tauri* may be interesting alternative model species for CK2.

### CK1, CK2 and the circadian clock

**Figure 9:**
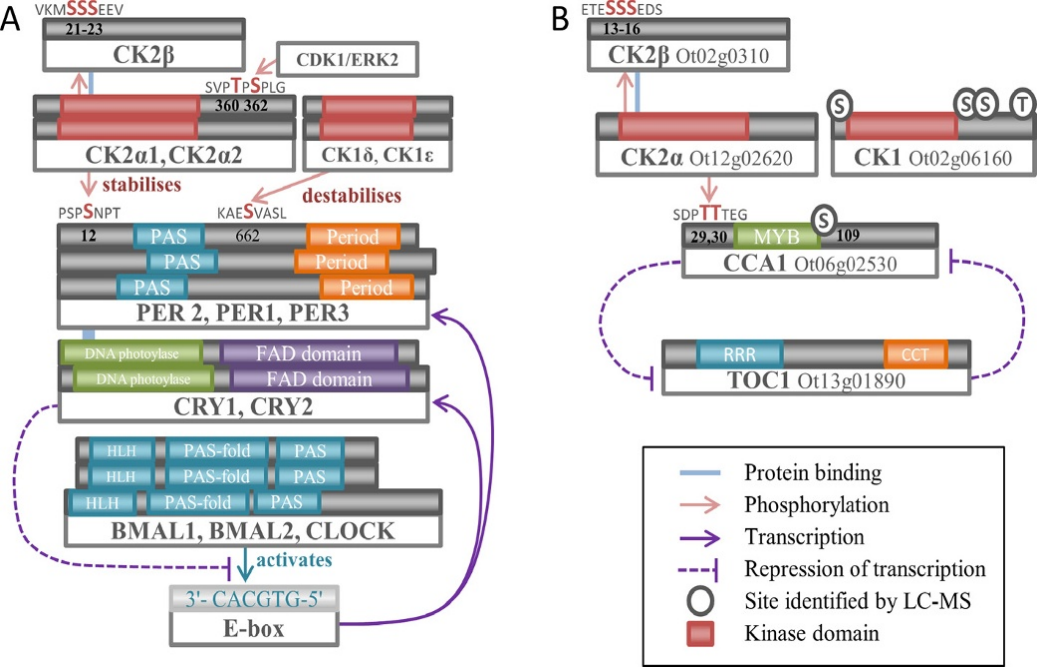
**The core transcriptional circadian clock of (A)** ***H. sapiens*** **and (B)** ***O. tauri*** **.** CK1 and CK2 regulate both clocks though PER and CCA1 phosphorylation. The downstream core clock however differs: in *H. sapiens*, CK2 has been shown to stabilise PER2 and promote its nuclear accumulation [121]. PER2 in turn stabilises Cryptochrome (CRY)1/2 in complex, which represses the transcriptional activation of PER1/2/3 via CLOCK/BMAL1/2, creating an oscillating feedback loop. In *O. tauri*, CK2 phosphorylates the morning-expressed CCA1 (by similarity with *A. thaliana*, which represses the transcription of the evening-expressed TOC1. TOC1 in return represses the transcription of CCA1, again creating an oscillating feedback loop. PAS – Period – FAD – HLH – helix-loop-helix domain MYB – RRR – response regulator receiver domain CCT – CCT motif.

CK2 is one of the few conserved components of the eukaryotic circadian clock [28, 103, 113, 114], where it fine-tunes period length and amplitude by dynamic modification of core-clock proteins. In *A. thaliana*, CK2 phosphorylates Circadian Clock-Associated 1(CCA1) and Late Elongated Hypocotyl (LHY) and over-expression of the CK2 regulatory subunit CKB3 shortens the period of these clock genes, accelerating plant flowering time [115, 116]. CK2 is involved in temperature compensation of the clock in fungi and plants, which allows for robust timekeeping [117, 118]. Within *O. tauri* we have identified CK2 motifs in CCA1 which are conserved (Figure 9, Additional file 4: Figure S3E) with the observed *A. thaliana* sites [115, 116], which indicate that this interaction may be retained. We also observed an S109 phosphorylation, in a Chlorophyta specific region, at the C-terminus of the MYB DNA binding domain which conforms to an E-X-S CK2 substrate motif. In metazoa, CK2α directly phosphorylates PERIOD (PER). Mutation of CK2 delays the nuclear import of PER and lengthens circadian period [119] (Figure 9). A mutation in CK2β also lengthens period [120]. CK2 regulates the mammalian clock by binding and phosphorylating the clock protein PER2 at S/T residues in the N-terminus, thereby stabilising the protein and promoting nuclear accumulation of PER2 [121]. Diminished PER2 stability can have opposing effects on the circadian period by affecting the timing of when PER2 is accumulated in the nucleus – accelerated and prolonged nuclear import speeds up and slows down the clock, respectively.

A naturally-occurring short circadian clock period phenotype of 20 hours was first observed in Syrian hamsters (*Mesocricetus auratus*) and attributed to the *tau* mutation in CK1ϵ [122]. The *tau* mutation increases PER1 and PER2 phosphorylation, increasing proteasomal degradation, and shortening the circadian period [123]. CK1 control of the clock is exemplified by familial advanced sleep phase syndrome, a condition associated with early sleep time followed by early morning awakening, whereby a mutation to human PER2 or CK1δ advances period [124–126]. Mutations to *D. melanogaster* CK1δ lengthen period, suggesting differences in the regulation between mammalian and insect clocks [126]. Until recently, CK1 has not been implicated in plant clocks. There are early indications for a functional role for CK1 in the *O. tauri* clock [31, 32], however the exact targets of CK1 are unknown.

### Smaller kinase families in *O. tauri*

Additional file 5: Table S2 shows that the remaining protein kinases span a wide range of families, which are discussed in Additional file 8: Figure S6: two of the highly-conserved RIO family, a Polo-like kinase (PLK), an Aurora kinase, BUD32, five STN-like kinases, two BUB1-like kinases, Haspin, and two HKs. The *O. tauri* kinome therefore comprises a suitably diverse set to represent many of the known protein kinase functions in eukaryotes.

## Conclusion

We identified 133 gene loci encoding catalytic protein kinases in *O. tauri*, constituting a small kinome of a similar order to *S. cerevisiae* (130 genes). As a photosynthetic model for core signalling, it is nearly ten-fold smaller than the *A. thaliana* kinome [4]. Comparing *A. thaliana, S. cerevisiae* and *H. sapiens* sequences, we found *O. tauri* kinases were frequently more closely related to the *H. sapiens* sequences than were the *S. cerevisiae* orthologs (the PIKK kinases are one exception). Thus genome reduction in *O. tauri* has not led to divergence in its kinome, in contrast to the minimal kinomes of parasitic species. DNA-PK is present in *O. tauri* and *H. sapiens* but absent in *A. thaliana*; PKG in *O. tauri* is also closer to the metazoan and fungal sequences than the closest *A. thaliana* sequences; several other components are conserved in exemplar signalling pathways (genes, phosphorylated amino acids and binding motifs), such as S6K activation *via* the TORC1 pathway. Together with other conserved components that are otherwise absent or poorly conserved in much of the green lineage, such as the cell cycle phosphatase CDC25 [127], our kinome survey indicates that *O. tauri* is a reduced but representative laboratory model species for signalling research, which incorporates many eukaryote-wide signalling components.

## Methods

### Identification of OrthoMCL ortholog-groups

We obtained ortholog groups assignment for *H. sapiens*, *S. cerevisiae* and *A. thaliana* from the OrthoMCL version 5 database and used the proteome upload service [128] to annotate the *O. tauri* peptide sequence from BEG (Additional file 3: Figure S2), which we supplemented with our corrected gene models.

### Identification of kinases in *O. tauri*

In an approach similar to Vilella *et al.* [129] we clustered all sequences from *H. sapiens*, *S. cerevisiae*, *A. thaliana, O. lucimarinus*, and *O. tauri* into related protein families. For *O. tauri* we used the December 2006 peptides sequences, and for *O. lucimarinus* we used the JGI November 2011 peptide sequences, both retrieved from BEG (Additional file 3: Figure S2). The TAIR (version 10) representative gene models for *A. thaliana* and Uniprot reference proteomes for *S. cerevisiae* and *H. sapiens* were retrieved in November 2012. We searched all proteins, against all proteins, using the NCBI blastp tool (version 2.2.25; BLOSUM62) with an e-value cut-off of 0.01. We calculated the BLAST Ratio Score (BSR) for each hit found [130] and we retained best-reciprocal hits and BSR scores greater than 1/3. We created a distance matrix using the BSR scores and applied the Markov Cluster (MCL) algorithm, (version 12–068), with inflation values 1.1 and 1.4. We extracted groups of kinases and phosphatases from the subsequent clusters. We searched for kinase and phosphatase catalytic domains with the hmmsearch algorithm (HMMER 3.0, GA cut-off) [131] using the models provided in Pfam-A (January 2013) [132]. We also ran a sequence similarity search with an e-value cut-off of 0.07, using the blastp algorithm (BLOSUM62), from *O. tauri* against the PlantsP database. All these results were manually curated to extract all candidate kinases and phosphatases into a database.

### Constructing alignments and phylogenies

Kinase alignments for each family in *O. tauri* were constructed by whole sequence alignment of protein sequences to whole families of proteins. The KinBase database was used as a source of *S. cerevisiae* [133] and *H. sapiens* [6] kinases annotations (Additional file 9) and family. The PlantsP [37] database provided *A. thaliana* kinase annotations. We aligned sequences using MAFFT [134] version 6 within JalView [135, 136]. We used the high quality global alignment algorithm G-INS-i, with BLOSUM62, 2-tree rebuilds, gap open and extension penalties of 1.53 and 0.12 respectively, and a limit of 1,000 iterations. Poorly aligned sequences were manually removed from the alignment. For editing alignments of more than 8 sequences we used guidance version 1.3.1, with the same MAFFT parameters previously described, and 100 guidance bootstraps [137]. We retained columns with a confidence value greater than 0.93, and sequences with a confidence value above 0.6. Columns with gaps were excluded. Inference of phylogenetic trees on the conserved alignment columns was performed using a Maximum Likelihood (ML) approach. Phylogenies were built with RaXML version 7.2.8 [138]. We used a γ model of evolutionary rate heterogeneity combined with an estimation of the proportion of invariant sites. Amino acid replacement scoring was determined using the WAG matrix [139]. Support for branches on the ML tree was evaluated using bootstrap analysis, using the frequency-based criteria (FC) parameter to determine the number of iterations. We used the FigTree version 1.4.0 tool for the visualisation of trees.

When alignments of *O. tauri* proteins contained gaps, extended inserts which were not found in other species, or poor alignments, we investigated and where appropriate corrected underlying gene models. Where gaps where present in the *O. tauri* genomic sequence, we used the closest gene from *O. lucimarinus* to infer the gap sequence, when there was a high degree of conservation in the adjacent region (Additional file 3: Figure S2).

### Phosphorylation-site identification by tandem mass spectrometry

Protein extract from *O. tauri* cells was prepared in a similar manner as described previously [26], with the digestion performed on 300 μg protein extract. Peptides were cleaned by reverse phase and phosphopeptide enrichment and LC-MS analysis were performed as described previously [26].

All multi-charged ions (2+, 3+, 4+) were extracted from each LC-MS file and MSMS data was searched using MASCOT Version 2.4 (Matrix Science Ltd, UK) against the *O. tauri* subset of the NCBI protein database (12/01/2011; 8,726 sequences) using a maximum missed-cut value of 2, variable oxidation (M), N-terminal protein acetylation, phosphorylation (S, T, and Y) and fixed carbamidomethylation (C). Precursor mass tolerance was 7 ppm and MSMS tolerance 0.4 amu. The significance threshold (p) was set below 0.05 (MudPIT scoring). A minimum peptide cut off score of 20 was set, corresponding to <3% global false discovery rate (FDR) using a decoy database search.

Ambiguous sites were confirmed by cross-referencing (by sequence, charge, and quantity of residue modifications) with most probable site predictions from MaxQuant (version 1.0.13.8 in singlet mode, same Mascot settings) [140].

### Availability of supporting data

All sequences and supporting data are included as additional files and are available at http://hdl.handle.net/10283/563.

## Electronic supplementary material

Additional file 1: Table S1: Phosphorylated peptides identified by LC-MS. A compilation of 3,994 uniquely identified phosphorylation sites in *O. tauri*. These describe phosphorylations of 2,214 peptide sequences, which correspond to 1,252 proteins. (XLSX 699 KB)

Additional file 2: Figure S1: Protein domain diagrams with phospho-sites. Domain diagrams for *O. tauri* protein kinases, grouped according to protein family. PfamA protein domains were detected by hmmr3 (PfamA downloaded Oct-2013). The locations of experimentally observed phosphorylation sites are annotated above the domain track. (PDF 66 KB)

Additional file 3: Figure S2: Sequences for novel and adjusted *O. tauri* gene models. We list the default sources of protein sequences and *O. tauri* sequences that were novel, derived from adjusted gene models, or have been patched with orthologous sequence from *O. lucimarinus*. (DOCX 119 KB)

Additional file 4: Figure S3: Evidence for conservation of phospho-site regions in *O tauri.* Sequence alignments for conserved phospho-site regions implicated in the phosphorylation of (A) MAP2K (Ot04g04050) by MAP3K and GSK3, (B) S6K (Ot07g02590) by GSK3, (C) S6K (Ot07g02590) by TORC1, (D) S6K (Ot07g02590) by PDK1 and (E) CCA1 (Ot06g02530) by CK2. Black framed amino acids specify a conserved phosphorylation site, and red framed residues show the conservation of a pre-primed phosphorylation site. (DOCX 160 KB)

Additional file 5: Table S2: Kinase and phosphatase classifications in *O. tauri.* The description and classification of 133 *O. tauri* protein kinases and 34 phosphatases based on sequence phylogeny and domain structure. (XLSX 17 KB)

Additional file 6: Figure S4: Kinase similarities between *O. tauri* and other model organisms. Box and whiskers plot that describes for each species the distributions of the Blast Score Ratio (BSR) similarities of *O. tauri* protein kinases against the best hit in the given species. BSR scores for each *O. tauri* kinase are calculated against the best hit (highest score) found in *H. sapiens*, *S. cerevisiae*, *A. thaliana*, and *O. lucimarinus*. Red crosses show the outliers, whiskers indicate the extremes of the distribution (excluding outliers). Boxes show the upper and lower quartiles, dissected by the red median line. The blue dot indicates the mean. Notches, indicating the 95% confidence interval, were calculated from 100,000 bootstraps. Table one of Additional file 6 gives the significance of the differences between species for the mean Blast Score Ratio (BSR) similarities of kinases. (DOCX 64 KB)

Additional file 7: Figure S5: Phylogenies of the main kinase families. Phylogenies for the (A) CMGC, (B) AGC, (C) CAMK, (D) STE, and (E) CK1 protein kinase families. *H. sapiens* and *S. cerevisiae* kinases are labelled according to their KinBase identifiers. *A. thaliana* kinases are labelled with AGI accessions. Accessions for *O. tauri* sequences refer to the BEG gene models except where we have altered a gene model (Additional file 2: Figure S2). Bootstrap confidences are assigned to edges. A broken-line edge indicates a bootstrap confidence of less than 40. (PDF 152 KB)

Additional file 8: Figure S6: Discussion and phylogenies of further kinase families. Phylogenies and descriptions for the (A) TKL, (B) BUD32, (C) RIO, (D) STN, (E) SCYL (F) ABC1, and (G) Histidine Kinases families. (DOCX 692 KB)

Additional file 9: Table S3: Sequence accessions for phylogenies. RefSeq and UniProt protein sequence accessions for the *H. sapiens* and *D. melanogaster* sequences, referred to by gene name in the phylogenies. (XLSX 13 KB)
