# Supplementary figures and images for: The reduced kinome of Ostreococcus tauri: core eukaryotic signalling components in a tractable model species
Source: BMC Genomics. 2014 Aug 2;15:640. doi: 10.1186/1471-2164-15-640 (PMC4143559; doi:10.1186/1471-2164-15-640)

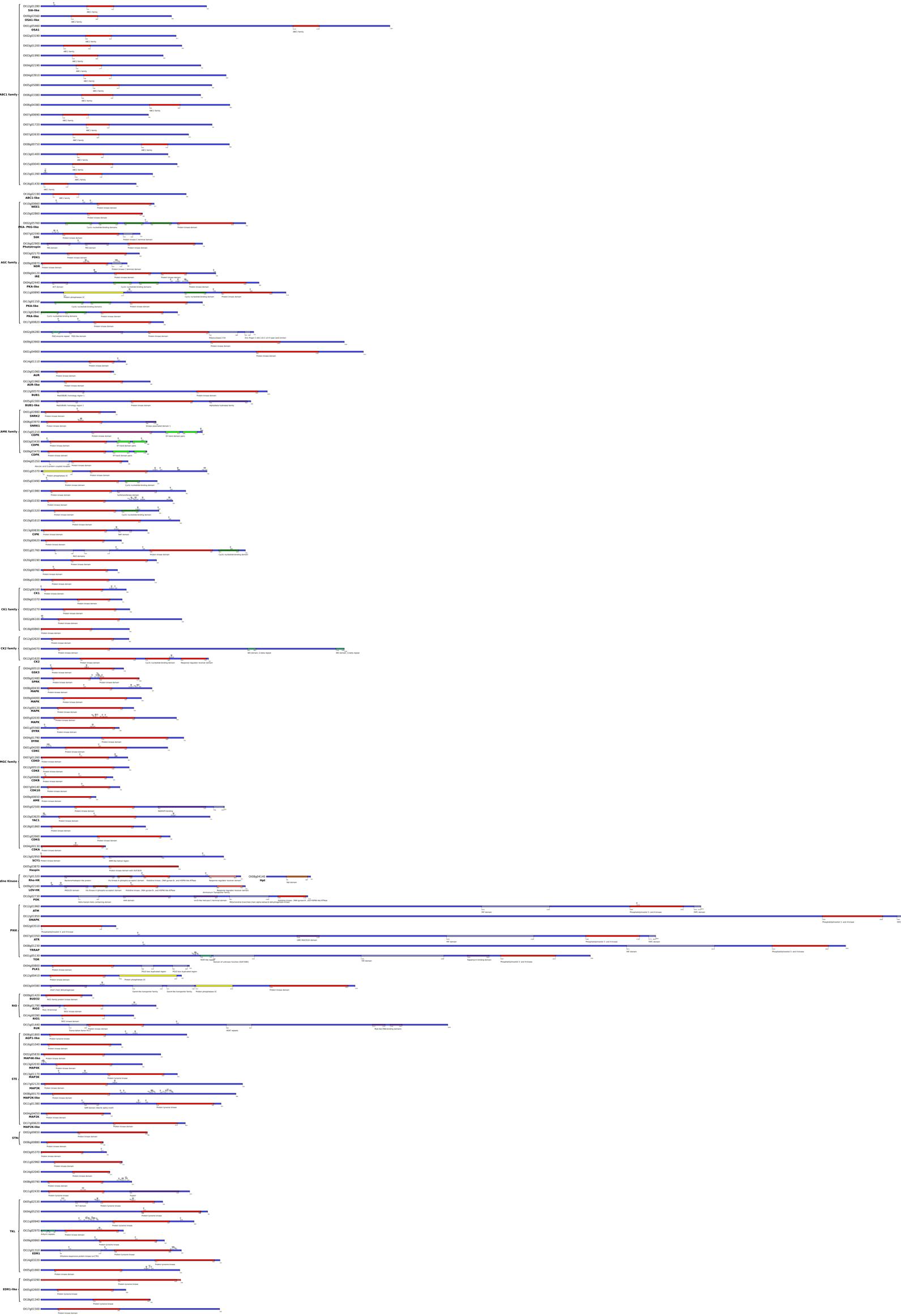

Supplement: Supplementary file 2 — Additional file 2: Figure S1: Protein domain diagrams with phospho-sites. Domain diagrams for O. tauri protein kinases, grouped according to protein family. PfamA protein domains were detected by hmmr3 (PfamA downloaded Oct-2013). The locations of experimentally observed phosphorylation sites are annotated above the domain track. (PDF 66 KB) [file 12864_2014_6366_MOESM2_ESM.pdf]

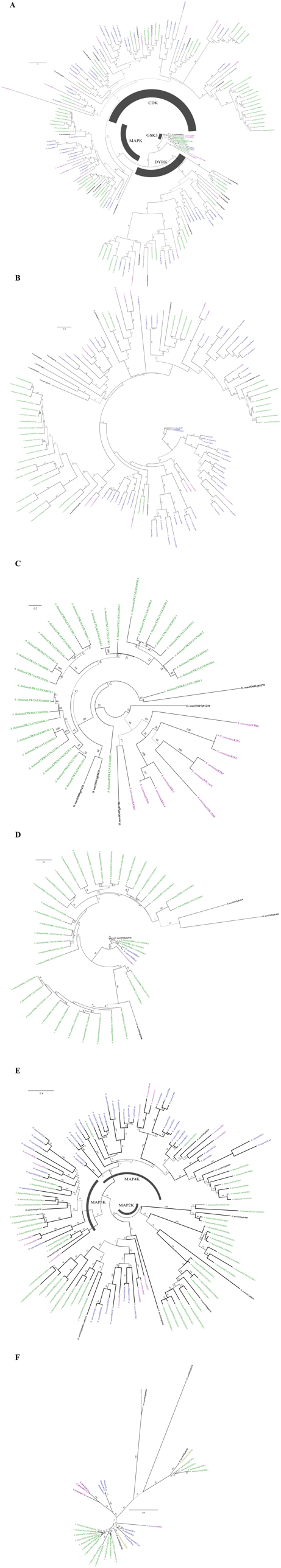

Supplement: Supplementary file 7 — Additional file 7: Figure S5: Phylogenies of the main kinase families. Phylogenies for the (A) CMGC, (B) AGC, (C) CAMK, (D) STE, and (E) CK1 protein kinase families. H. sapiens and S. cerevisiae kinases are labelled according to their KinBase identifiers. A. thaliana kinases are labelled with AGI accessions. Accessions for O. tauri sequences refer to the BEG gene models except where we have altered a gene model (Additional file 2: Figure S2). Bootstrap confidences are assigned to edges. A broken-line edge indicates a bootstrap confidence of less than 40. (PDF 152 KB) [file 12864_2014_6366_MOESM7_ESM.pdf]
