# Supplementary material for: The reduced kinome of Ostreococcus tauri: core eukaryotic signalling components in a tractable model species
Source: BMC Genomics. 2014 Aug 2;15:640. doi: 10.1186/1471-2164-15-640 (PMC4143559; doi:10.1186/1471-2164-15-640)
Supplement: Supplementary file 3 — Additional file 3: Figure S2: Sequences for novel and adjusted O. tauri gene models. We list the default sources of protein sequences and O. tauri sequences that were novel, derived from adjusted gene models, or have been patched with orthologous sequence from O. lucimarinus. (DOCX 119 KB) [file 12864_2014_6366_MOESM3_ESM.docx]

Sequences and adjusted gene models

The following supplemental information describes the protein sequence data used in the phylogenetic analysis presented in this paper. Table 1 describes the default source of protein sequences. Any modifications to *Ostreococcus tauri* sequences are also described below. Sequences information is presented for novel gene models, existing gene model alterations, and inference of gaps in *O. tauri* sequences based on orthologous sequences in *Ostreococcus lucimarinus*.

Table 1 Locations of protein sequence data used in this paper.

| Species | Database | URL | Version |
| --- | --- | --- | --- |
| *Ostreococcus tauri* | BEG | https://bioinformatics.psb.ugent.be/gdb/ostreococcus/O.tauri | Dec-2006 |
| *Ostreococcus lucimarinus* | UniProt | http://www.uniprot.org/uniprot/?query=organism%3a436017+keyword%3a181&force=yes&format=fasta | Nov-2012 |
| *Saccharomyces cerevisiae* | KinBase | http://kinase.salk.edu/kinbase/FastaFiles/ Bakers_Yeast_kinase_protein.fasta | Nov-2012 |
| *Arabidopsis thaliana* | TAIR | ftp://ftp.arabidopsis.org/home/tair/Proteins/TAIR10_protein_lists/ | TAIR 10 |
| *Homo sapiens* | KinBase | http://kinase.salk.edu/kinbase/FastaFiles/Human_kinase_protein.fasta | Nov-2012 |

# Sequences of novel gene models

>*Ostreococcus tauri*|Ot03gTAP42 Chromosome 3 - 14703: 15716 sequence

ATGACGCGAGAACTCCCGCTCGCGGCGTTGTTCGCGCGCGCCGATCGCGCGGACGCGTCGGTGGAGACGTGTGAGGCGTTCAGGGACGCCGTGCGCGCGACCGGGGGACTGGGACGGGCGGAGACGTTGGATGATTTGGATCGCATCGGGCTAAAGCTTGCGCTGGGGCATCGATTGGCGGGACGCGCGCACGCGCGATGCGGACGCGATGGCGTCGAACGGGCGATCGATGCGTTGGAGGCATTCGTCTCGCTCGTGCGCGAGCTCGATGGGGTGCCGCGGGAGGAGATGGCGGCGTGCGAGGGAGAGGAAGAGGGGACGTCGCGGCGGGCGCGGGGCGGAGACGGACGCGCGGATAAAGTGCGCAGATTTAAGGCGAAAAGGCGGTGTGAGAAGAGAATGGAGGAGATTGAGGAGATTTTGAAGCGACGATCGAGGGTGGAGGAGGAATCTGGGAGTGAATCCGCGTCGGAGGAGGATGAGACGGATCACGACGCGCTCGAGCGGGAGTATTGGATGAAACGCATCGAGCTCGAGACGTACGAGACTTTGGACGAACTCCCGAGCCTGAGAATGGAGAGAGAGATGTTTTTGAGACGGGATGAGCTCGAGGAGGCGCGACGGGCCGAGCGAGAGCGCATGGACGCGGATGAGCGCGTCGGACGCGACGCCAGGATCGAGACGTACACGATCGAGCGCGAGGACGTGAACGCGCTCGCGGGGCCGTCCATTCTCACCGCCGATCCGCGCACGAGATTCCGAACCGAGGTCTTCAGACCCACGGTGGCGTTACCCACGATGACGGTCGAGCAGTTCGGCGAGATCGAGCGTCGGGAGATGCTCGAACGCGAGCTCCGCTCGGCGGAGCGCGAGCTCGAGCGCGCCGCCATCCGCGCCGCCAAAACAGAGGAACAAATCGAGGAAGAAGAGCTCGCCGAGACGCGTCGATGGGACGCGTTCAAGGACGACAATCCCTACGGCTCGGGGAACTCCCGGCTCCGGCCGTGTTCGTAG

>*Ostreococcus tauri*|Ot03gTAP42 Chromosome 3 - 15342: 15716 MW: 14389.101 05/06/06 sequence

MTRELPLAALFARADRADASVETCEAFRDAVRATGGLGRAETLDDLDRIGLKLALGHRLAGRAHARCGRDGVERAIDALEAFVSLVRELDGVPREEMAACEGEEEGTSRRARGGDGRADKVRRFKAKRRCEKRMEEIEEILKRRSRVEEESGSESASEEDETDHDALEREYWMKRIELETYETLDELPSLRMEREMFLRRDELEEARRAERERMDADERVGRDARIETYTIEREDVNALAGPSILTADPRTRFRTEVFRPTVALPTMTVEQFGEIERREMLERELRSAERELERAAIRAAKTEEQIEEEELAETRRWDAFKDDNPYGSGNSRLRPCS

# Sequences for proteins with adjusted gene models

The most critical gene model adjustment was S6K (Ot09g03470). The existing gene model contained a gene fusion and a frame-shift near the activation loop, which would almost certainly result in a non-functional protein. We compared the genomic sequence of Ot09g03470 to *O. lucimarinus* (OSTLU_3119) and found an extremely high level of conservation at the nucleotide level. We suggest that the two deletions in Ot09g03470, near the activation loop, are the result of sequencing errors as it is likely they would result in a non-functional protein. We were unable to find any reasonable splicing solutions which would accommodate these deletions and retain a functional protein. If these deletions were genuine we would not expect such a high degree of conservation in this region, and in the region of the potential intron (if the gene was spliced to the correct frame). There is a single copy of S6K in the *O. tauri* genome. It is therefore unlikely, given the high degree of conservation, that Ot09g03470 is a pseudogene. The following alignment shows the conservation of S6K in *O. tauri* and *O. lucimarinus.* The proposed sequencing errors, which resulted in two deletions, are highlightd.


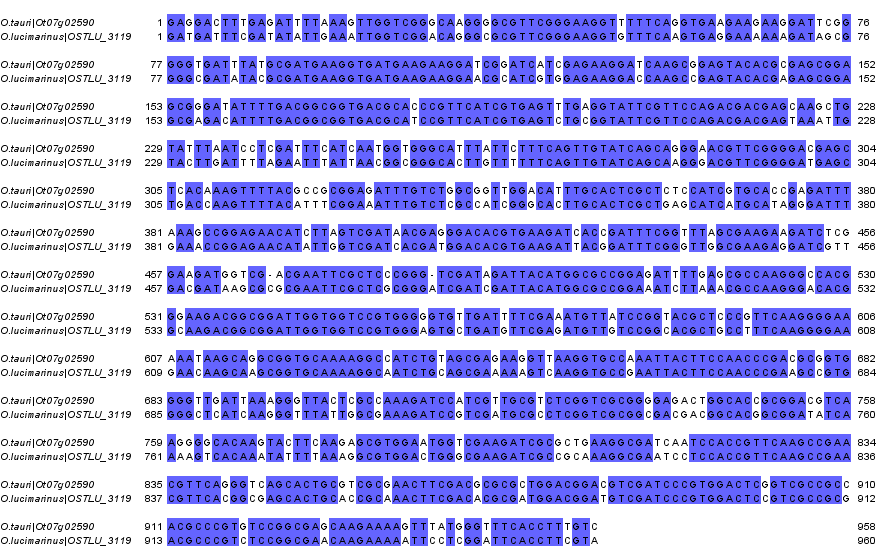


The following nucleotide sequence is the corrected sequence for the *O. tauri* S6K (Ot07g02590), based on the assumption that the two sequencing errors we inferred previously are correct. We have supplemented the nucleotides from *O. lucimarinus* S6K (OSTLU_3119) at these deletion sites, based on their alignment.

>Ot07g02590 a proposed corrected sequence based on identification of potential sequencing errors. The two inserted bases (bold and underlined) are proposed from the alignment with O. lucimarinus

ATGACGACATCCGCGAGCGACGCCGAGGCGCGCCGCGCGTGGGCGCTCAGTGGACTCGGATACGCGATCGCGGAGGGCGATGGGACGCGCGCCGCGGCGGCGGGGACGCGAAAGATGGATGGGTTCGACGTGCGAGCGACGAGCGATGGGACGGAGACGCCGACGTTGAAGCGACAACACAGCTCCGGGGTGAGCATGCTCAGCGCGAACCTGCGACGGATCGATAGTGGGTTGTTGCTGGACGAGACGCCGAGCGCGAGCGGGGCGCGAGAGACGAAAAAGTTGTCGCCGGAGGACTTTGAGATTTTAAAGTTGGTCGGGCAAGGGGCGTTCGGGAAGGTTTTTCAGGTGAAGAAGAAGGATTCGGGGGTGATTTATGCGATGAAGGTGATGAAGAAGGATCGGATCATCGAGAAGGATCAAGCGGAGTACACGCGAGCGGAGCGGGATATTTTGACGGCGGTGACGCACCCGTTCATCGTGAGTTTGAGGTATTCGTTCCAGACGACGAGCAAGCTGTATTTAATCCTCGATTTCATCAATGGTGGGCATTTATTCTTTCAGTTGTATCAGCAGGGAACGTTCGGGGACGAGCTCACAAAGTTTTACGCCGCGGAGATTTGTCTGGCGGTTGGACATTTGCACTCGCTCTCCATCGTGCACCGAGATTTAAAGCCGGAGAACATCTTAGTCGATAACGAGGGACACGTGAAGATCACCGATTTCGGTTTAGCGAAGAAGATCTCGGAAGATGGTCG**C**ACGAATTCGCTCCCGGG**A**TCGATAGATTACATGGCGCCGGAGATTTTGAGCGCCAAGGGCCACGGGAAGACGGCGGATTGGTGGTCCGTGGGGGTGTTGATTTTCGAAATGTTATCCGGTACGCTCCCGTTCAAGGGGAAAAATAAGCAGGCGGTGCAAAAGGCCATCTGTAGCGAGAAGGTTAAGGTGCCAAATTACTTCCAACCCGACGCGGTGGGGTTGATTAAAGGGTTACTCGCCAAAGATCCATCGTTGCGTCTCGGTCGCGGGGAGACTGGCACCGCGGACGTCAAGGGGCACAAGTACTTCAAGAGCGTGGAATGGTCGAAGATCGCGCTGAAGGCGATCAATCCACCGTTCAAGCCGAACGTTCAGGGTCAGCACTGCGTCGCGAACTTCGACGCGCGCTGGACGGACGTCGATCCCGTGGACTCGGTCGCCGCCACGCCCGTGTCCGGCGAGCAAGAAAAGTTTATGGGTTTCACCTTTGTCTCAAACTTCATGGAGGAAGCGGCCGTCAGGCTCGCATCGACGACGATTTATCCTCCGCGAGCGCGTCCGAGGA

The following amino acid sequence is the translation of our proposed *O. tauri* S6K (Ot07g02590) sequence.

>*Ostreococcus tauri*|S6K|AGC|Ot07g02590 We have removed an incorrect gene fusion and resolved a frame shift error which affected the activation loop (targeted by PDK1). We speculate there is a sequencing error (as there is no deletion in *O. lucimarinus*, which is highly conserved at the genomic level).

MTTSASDAEARRAWALSGLGYAIAEGDGTRAAAAGTRKMDGFDVRATSDGTETPTLKRQHSSGVSMLSANLRRIDSGLLLDETPSASGARETKKLSPEDFEILKLVGQGAFGKVFQVKKKDSGVIYAMKVMKKDRIIEKDQAEYTRAERDILTAVTHPFIVSLRYSFQTTSKLYLILDFINGGHLFFQLYQQGTFGDELTKFYAAEICLAVGHLHSLSIVHRDLKPENILVDNEGHVKITDFGLAKKISEDGRTNSLPGSIDYMAPEILSAKGHGKTADWWSVGVLIFEMLSGTLPFKGKNKQAVQKAICSEKVKVPNYFQPDAVGLIKGLLAKDPSLRLGRGETGTADVKGHKYFKSVEWSKIALKAINPPFKPNVQGQHCVANFDARWTDVDPVDSVAATPVSGEQEKFMGFTFVSNFMEEAAVRLGTAGSRTAAEEDIDDDLSSASASED

The following amino acid sequences are the result of corrections to the underlying gene-models based on bioinformatics analysis. The corrected sequences are used for all phylogenies presented in this paper. Amino acid sequences that are derived from *O. lucimarinus* have been underlined.

>*Ostreococcus tauri*|Ot09g03470

MGGDAGTRSATTTRATVRILGDAASAVDVREAYAFGVELGRGQFGEVREVVDTSTGRALACKSISKRGLKSESSREMVRNEVRIMHHLAGIRRLVSVHEDATHVHLIMEKLDGPEWFDAITEQFDEAPYSEAEAAAQFRNVARTVEYLHAMDVMHRDLKPENFVLKSKEKDSPICAIDFGLSTFFLPDQEFTELVGSPYYVAPEVMGYKYSNEADVWSCGVILYILLSGVPPFWGSSEKQIFDEIKRYKTGATSLDFASEPWPSVSDAAKELVQGMLTVNRKDRMTIEDVLSHPWLADPGVAPKTALDNIVLQRFKNFAGMDKFKRFGLHAMARSMPEEEVIGLGVMFKELDKDKSGKITIVELRQGLKLQSAEAAAQLEEVVASVDLDGSGDLDYEEFIVATIARSKRESRAAVQRAFDFFDIDGDGSITADEFQRALESLSPVERTNLGDVNELLAAADTNGDGVIDFDEFMAAMSSDTDAR

>Ostreococcus_tauri|AGC|PDK1|Ot03g02170 Later start site and changes to splicing

MIQEQGELVTLPTKLDYFTDVHRSKDTRTIVELPPKEPSEEEDPPQGFARIFACCFAPKILCFTKFPSQPVVAVSVNPPSRLGRSPGSAHAFVTARRLACRLAIERARTPPRTPPFEISDFVTVDVIGEGSYSDVREVFLSSRPSERYALKVMDKAHIVRESKSRYVATERTLLAGRLRECEHVARLMFTFQDTYSLYMGFELCPGGDLFWQLKRSEEGVMEETKVVFYVSEVLVAVQDCHARGVVHRDVKPENVLIDASGHVKICDFGSALDLRHEVTSALTALASEKRCASFVGTAEYVAPEILDGCEETTTAVDLWSIGIMTFQLLTGRVPFKGKTEYLTMQERTSTRLSANISESAKDFIDSLLTRDPKKRLGYENETSIRNHPFFAAVDDWSELRSREAPRVLTATGVGSDATVTDSECDHDDGTDTDDEWRARVNAAAAALDAL

>*Ostreococcus tauri*|CK1|Ot02g06160

MSASNGGLELRVGNKYRLGRKIGSGSFGDIYLGTHIKTNEEVGIKLESCKTKHPQLLYETRLYKILQGGTGVPNVRWYGIEGDYNIMVMDLLGPSLEDLFNFCNRKLSLKTVLMLADQLVSRIEYVHSKSFIHRDIKPDNFLMGLGKRANQVNIIDFGLAKKYRDPKTHLHIPYRENKNLTGTARYASINTHVGIEQSRRDDLESLGYVLIYFLRGSLPWQGLKAATKKQKYEKISEKKMTTPIEVLCKGFPPEFVTYFQVVRSLRFSDKPDYSYLRKLFRDLFIREGYQYDYVFDWTILKYQQTQALTRPTAGLGGQAQAGTSGDQGDQRDTLRRQTTLERQSSATRAAGGKEVLATERSSSFARAYERQRSSSRPLTSRDDDGGRAYQ

>Ostreococcus tauri|TOR|Ot01g05130

MCAIESEGEDDKDVCAEGSGGNGGEERGRGDFLGFRNSVHARVGAMIGSADTAKKMGGLRAIDQLIDVEFGEEVEKVRKFAEYVLEVMPPGGEERAQTKGEGARHPVDARNPLWGLPSAPQLIELMTAVIGRLVAHGGALTTDIVDSHVQRAVDLLETKEDNKEALSMKHYAAVRTLSELARNAPTIFNVHVQIVVVAIWSALRDPSLQVREAAVSALRDCLMVIEQRETRYRVQWYYSLYESCRVGLRSESSVEAMHGSLLALGELLRYTGEFMLSRYREVAQTIFRLQDCKASIIRRTIVLLIPKLAVFSPRRFAESYLVESCALILTTIRASSDSGAGFEALSQLADAMSHVINLSTISPEKADVGGVLLRYLPEVSAVMYDLFTTRARDQQGTPEALKCAGSLMNSLNEAWKPHFMNLMTPMFMGDLSEALVSGLDFVAKSSPELMPEIQMKLADSIESAISQGPETVTPNRKTVQLALRTMRTFPFEAVALLRAIKKNVVPYLSDSSAETRLEAALTCCSTLRLRVGSSKSATRAVEYIMAALIPVAVGDTDASIRSSLLSDFCRPCASIDSYLGQAKSLRALFLTVNDESVTIRMLGIELLGHLATRNPAYVLPALRAHMLQLLAELEFSSESLHREESAKVLAVLIRSCPRFVAPYLSAILNVLTATLRADEGGSSATSTSTRESMTALKGRLQRTGSSGSGALTSSIPATLRKRRDVTGREKAAVLGTIGELANVSGSEIKPFVQPLLALLLIALKSPSTRDEAVVTLGKLIESSGYVADPFTTHPQLLPQLLRILANERGIARNEVLRTLGCLGALDPYAHKANEIRLHGEGVLSADGVRGVRQASLKLAAGTGEDDILDEFLNNADGDDEIDVLPTLHLTSLSDEYYPTIALNSLLRILRDPTRTSHRLMVIRSIVYIFQVLDTGCVQYLPNVLPVMLNVIRTSDPSRREDMFVELATLVSVVKAHIRRYLTDIFELMHLTWDVTGMSKNTILLCEELSFALNDEFRKNLPTIIPSVVAALGEAERLRKYDLVPHILHALECFGVSLNDHLYLLMPPIMRLLKHSVISVPVEIKKCTLESLRRLLPIMNLSDQAALIVHSLTRVLDDERMELRMEALCVLTSLEVSLGRDYALFLPVVKRAVSKCGVNDPAFDEMVKRVESGNVLLYPDEELETSSVVATARKKPDIQRRLSVNQLAIRRAWESSQRSTKEDWLEWMRQLALDLLKSSPLPSLRACSELAQVQPNLARDLFCASFVSCWAELNESHREVLVRSLEAALGSPTIPPEIVSILLNLCEFMEHDEKPIPVDVRTLGMIAERSRAYAKALHYKELEFVTNPGACVAAIIAINNQLQLPEAARGVLVYAQENLSVVIKESWYEKLEQWDDALEAYKRKMDEVMHVTTPNAMEERREAMAGQLRCLNALAEWEEIHRISEIATKSNVDWDDYSIDVTTIATKAAWYLGDWEQMEVLTDKLERDQRSQGRLPTADRAELVSKHKTSVVNVTDCDFYKSIIAFRKGNQEEARTHLSAARDKLGTELPALVRESYDRSYRALIRAQQLTELDEVIEYAQLTAVASPAALQRQEVIRKMWRDRIYLVKRDVEVWQELLQVRSLVLPMSEETDIWLKFAALNRKQGRDRQSRRTLLRLLGYDPMLCAEETAGFGAGSGRPKVMFAFIKHLWHTGQKRQAFMRLQSLAHELRVTWENQRSSGQPPEVEHNKTVSRAFLKLGKWRWALTDCMNDETLTDVLMAFRTATAADQQWSKAWHHWALFNATAMEHFHRQGGASTQDAMQHVAPAISGFFRSIALGGGKTNAKQGGSLQDILRLLTLWFNHGGTAEVETALIEGFGHVSIDTWLAVIPQIVARIHTHVIPVRNLIYQLLIRVGRQHPQAVLYPLLVACKSQSTSRRAAANAILDNVRQHSALLVEQAQIVSLELIRVAIVWHEAWHEALEEASRLYFGEQNVDGMMAVLTPLHHILERQGAETLQEMGFVQNYGRELQEAHDLCQKYQMSKREEELNQAWDLYYHVFKRITKQLPTMTTLELQYVSPRLLNSRGLELCVPGNYISRATEQAKSEHGKDYGYLLKGHEDLRQDERVMQLFGLVNTLLNSNMTTQQKDLGIARYAVVPLSPNSGLIGWVPNCDTLHALIREYREAHKIPLNLEHRMMLAMAPDYDHLPLVNKVEIFMHAVENTSGGDLAHVLWLKSRSSEQWLERRTTYTRSLAVMSMVGYLLGLGDRHPSNLMIDRYSGKVLHIDFGDCFEASMYREKFPEKVPFRLTRMLVRAMEVSGIEGNFRSTCESVVTVLRDNKDSVMAMLEAFVHDPLINWRLLTHKIVPDNDENEVQEVALRSSSASDSGNSTPQPKSRGAGSKRPPSVGVVDTPSGPVPSSSSVIGVEAAHAMAAMSSSVRDHPSQAGSVLFRRTELIHALETYGMDDGANEALNERAVSVMQRMSAKLTGRDGEHHHVDTMLPDTVEKQVRRLVAEATSAENLSVSYVGWCPWW

>*Ostreococcus tauri*|ABC1|Ot04g02190

MVHGGTVIVRVSDRARKSGSGKARRRRGRRDPYDDLRGEFDKPKLLRFFRRRPLQVAGRLATIIRVGRRVIRQWKAQESWDPEERTRGAILREAMTRLGPVFVKIGQTLSQRPDLIGEEAADELKLLQQSNEPFPNALAWKTIVEDLEWDGPIAPNHPYRARNPDAEPLFAKFSEQPIAAASLGQVYKAKTWDGQDVAVKVQRPKVVRQVALDWTCWSLSLSTLKRLWGTTTELDVIADEVGQGVWQELDYTQEAAHMDEFNERHKWLGFVRAPHWLPEYTGPPGRARIITTEWINGQHIAALPPEKKLIMAQMAVEACVAQLIYTGFVHADPHEGNMMLDDNDMLVFLDFGLMSEVEPFIMEGFAKGIQHMISGNWEGLVLVFQEVGFTPKEGFLKRDDRSDTYKPATLEEMTKAVADTLSTEEGGQSRFGALATGLAKLSANFKFLTPPYIILLIRTFLTLEGIAEKADPDFNIYTAALPYAIRRAMAPSTPEGQIAMRNTFLNESDELRWDRIEELVLTDTVDEDDSKESADAENAEGAFGDSQALMARRSKEVVGRLLGSTEGVALRRVANSANTEKIVEYLSGPKGTALRAKSIRMLSRNLKDLWTARRFVRRATPQIDSLPVWPESDEARRIRERQDRAQKRALAFIFGTHMARLIRKPWLLVRALTVTAYIVVTAFVYALAVTVWDYLKWLNRFTPWGLVWRLASRGAPSGPS

SDALGSTSVP

*>Ostreococcus tauri*|DSP|Ot16g02430+A4S8J9

MPHVVDSDDDAPPPRAAWCRACGHDHAPGTTCGVCGHHRRVDDDLDDLPRPRLGHDEPIIEVIDKFLCLGAFEHTSKEDVLLACGIRTVMNAVPECTPCCSSKNICVFTAPVKRGCSTTLDLGETVKKLEALHARAMRADEAGAANRVLVYCMSGQSRAPSVAVAYLMYSERRRMDDALKALQRRYPRGYNGVTIKQKDVDELKMFEAELFPPGAS

*>Ostreococcus tauri|*Actin|Ot16g00370
MADEGEVSALVCDNGSGMVKAGFAGDDAPKAVFPSIVGRPRHHGVMVGMGQKDCYVGDEAQSKRGILTLKYPLEHGIVTNWDDMEKIWHHTFYNELRVDPAEHPVLLTEAPMNPKANREKMCQIMFETFNVPAMYVSIQAVLSLYASGRTTGIVLDCGDGVSHTVPIYEGYALPHAILRLDLAGRDLTDYMVKILTERGHSFTTSAEREIVRDIKEKLAYVALDFEEEMATSQSSSSIEKSYELPDGQMITIGSERFRCPEVLFQPSLIGMENPGIHETTYNSIMRCDVDIRRDLYANIVLSGGTTMFPGIADRISKELTALAPSSMKIKVVAPHERKYSVWIGGSILASLSTFQQMWIAKSEYNEAGPSIVHRKCF

>O.tauri|Ot12g01390 New Gene model Tubulin alpha subunit gene model 201785: 203233 MW: 49240.094

MREVISIHIGQAGIQTGNSCWELYCLEHGIQPDGQMPMDGSAGQEDDSFNTFFSETGAGKHVPRCVFLDLEPTVIDEVRTGAYRQLFHPEQLISGKEDAANNFARGHYTIGKEIVDVALDRIRKLADNCTGLQGFLVFNAVGGGTGSGLGSLLLERLSVDYGKKSKLGFTIYPSPAVSTAVVEPYNSVLSTHALLEHTDVAVMLDNEAVYDICRRSLDIERPTYTNLNRLLAQVISSLTASLRFDGALNVDVTEFQTNLVPYPRIHFMLSSYAPVISAEKAYHEQLSVAEVTASAFEPASMMAKCDPRHGKYMACCLMYRGDVVPKDVNAAVANIKTKRTIQFVDWSPTGFKCGINYQPPTVVPGGDLAKVQRAVCMISNSTAIAEVFSRLDHKFDLMYGKRAFVHWYVGEGMEEGEFSEAREDLAALEKDYEEVGASSAAEGFEDEY

>O.tauri|Ot08g03630 New Gene model Tubulin alpha subunit 601890: 603406 MW: 49658.703

MREVISIHIGQAGVQTGNSCWELYCLEHGIQPDGQMPSDKTIGASDDAFNTFFSETGAGKHVPRCIFLDLEPTVIDEVRTGAYRQLFHPEQLISGKEDAANNFARGHYTIGKEIVDLALDRIRKLADNCTGLQGFLVFNAVGGGTGSGLGSLLLERLSVDYGKKSKLGFTIYPSPQVSTAVVEPYNSVLSTHALLEHTDVAVMLDNEAVYDICRRSLDIERPTYTNLNRLIAQVISSLTASLRFDGALNVDVTEFQTNLVPYPRIHFMLSSYAPVISAEKAYHEQLSVAEVTNSAFEPASMMAKCDPRHGKYMACCLMYRGDVVPKDVNAAVASIKTRRTIQFVDWCPTGFKCGINYQPPTVVPGGDLAKVQRAVCMISNSTAIAEVFSRLDHKFDLMYAKRAFVHWYVGEGMEEGEFSEAREDLAALEKDYAEVGASSLSTGYEEGMDEY

>O.tauri|Ot05g05090 New Gene model Ubiquitin-conjugating enzyme 800704: 801366 MW: 17131.406

MSDKDKAKSLSNSARRIQKELAEISLEPPTNCSAGPKGDNLYEWVSTIVGPSESPYSGGVFFLDIQFPPDYPFKAPKVTFRTRIYHCNVNSSGQICLDILKEQWSPALTISKVLLSICSLLTDANPHDPLVGSIAQQFLNDKENHDKTAQEYAQG

>*Ostreococcus tauri|*SNRK|Ot06g03970+A4RZA4 (NB incorrect n terminal sequence removed before genomic gap)

MEGGEDSGTHASGGASRGQAEIYLPNYRIGKTLGIGSFGKVKVAEHVLTGHKVAIKILNRKKIKAIDMEEKVRREIKILRLFMHPHIIRLYEILETPHDIFLVMEYVKSGELFDYIVEKGRLGENEARHFFQQIISGVEYCHRNMVVHRDLKPENLLLDSRNNVKIADFGLSNVMRDGHFLKTSCGSPNYAAPEVISGKLYSGPEVDVWSCGVILYALLCGSLPFDDESIPNLFKKIKGGIYTLPSHLSPGARDLISRMLFVDPLKRITMAEIRQHPWFVVHLPRYLVVPPQTQISQTSNLDGETLDMVVNLGFEREPLISALQHQVRNKATVTYYLLLDNRRNIYGGYLGAEYDDAEMMAEHNNMRGHGVASALGVRRPSQIQSHMMQTRLVAEQRWMLGTASKMGAQDVINELLRVLRAMNIAWKKNGPYNMKCMCVFHPDNDGEMDAGDYGESDAMEADAGDVSMATRSHAPGNAREPSVLKFELQVYKLPNDRLMVDYQRVEGGVMVALDFAAKLMTFMNLQ

>*Ostreococcus tauri|CMGC|*Ot13g01150

MDTANGASGPKGVGGRDDADENARIRARARRVSEPESASEALDGVALFRELKRDARAALCGSVVECEYERGDVIVRKNEVGTHFYVVAHGCVRVDASEVGTRTGDYAGEASGEDDERRRHESRTRESSSSEREDASPMGGIALSYKLLYPGDTFGEVALVRKCPRTATVIAESSSVKLWALDRATFQETVKRTTFERRCRLTSYLDAVEVFRESLDARTMSMLVDALREVTFESGHVIVSPDDVYEEESSKFYVVIDGEAVVYVDDGSKAEVNRLRRGDYFGELSLIHKTSPTAMVIAVGQVSALTLDAESFYRMIGEKTVKLMARRIDSYTHTANKAERKLSKSCLSRCAGSFAALRGAMTSPFGWLSFDRRHAERQKISLNEIKRGRQTTSKKLSVDFDVHSSKDVLFVKEIGSGMLGSVFIAKTLNTQETCCVKVMHKWKIAHLDQEKNVCRELEIMKRFDSPFLTRAVGAFQDARSLYLLMDYLPGGDLFQLLVSGPYRGGRFPVNVAQFYAAEVFIALEYIHTEEYVYRDLKPENVLLDACGHVKLTDFGFCRHLLPGERAYTTCGTADYMAPEVMLAQGYDQLADYWSFGVLLYEMLVGYAPFASVSDGVRHRRIISSDLKFHSTYFSLTAKSLISRLCVVDVSKRLGSGARGSGDLKSHDFFRDLNWEDVTARRIDSPVRPPVRDRVSADVGSVGRHRSRELLRALDRACERERRERGAGSSAQSADFVGF

>*Ostreococcus tauri|*HSP90|Ot10g01300

MSEDTETFAFQAEINQLLSLIINTFYSNKEIFLRELISNSSDALDKIRFEGLTDKSKLESQPELFIHIVPDKTNNTLTIIDSGIGMTKADLVNNLGTIARSGTKAFMEALTAGADISMIGQFGVGFYSSYLVAEKVVVYTKHNDDEGYRWESQAGGSFTVTKDASASELGRGTKMVLHLKDDQLEYLEERRLKDLVKKHSEFISYPISLWTEKTTEKEVSDDEAEEEGEEEEGKITEIKDDEVKEKKTKKVKEVSHEWAIMNKQKPIWMRNPEEITKDEYAAFYKSLTNDWEEQLSVKHFAVEGQLEFKSVLFVPKRAPFDMFDGKKKANNIKLYVRRVFIMDNCEDIIPEYLGFVKGIVDSEDLPLNISREMLQQNKILKVIKKNIVKKCLEMFNEIAENKDDYTKFYEAFGKNLKLGIHEDAQNRSKIADLIRYYSTKSGEEQTSLKDYVTRMKEGQKSIYYITGESKKSVENSPFIEKLKKRGYEVLYMTDPIDEYAVQQLKEYDGKKLVSCTKEGLELDETEEEKKQKEEVAAQYENLCRLIKDILGDKIEKCIVSDRVVDSPCVLVTGEYGWSANMERIMKAQALRDNSMSSYMSSKKTMEINPDNSIMKELRKRADADKGDKTVKDLVLLVFETAMLTSGFSLDEPTTFGGRIHRMIKLGLSIDEDDAPVADDLPALEEEVDEGSRMEEVD

>*Ostreococcus tauri*|CCA1|Ot06g02530

MGDQGEAPSSNDTGDEATVTTNDATSDPTTTEGKAVKTRKPYTITKKRERWSDEEHALFVESLKKYGRAWKR

IEEYIGTKSAVQIRSHAQKFFAKLQKEQIVASGSEGSGSTRKRGADRSTSQSKRSKSSYATDINLEIPPARP

KKKPAHPYPRKATSQQPSGGSGERDNSGGTGKSSGTAQKWPTEASQEFIASTSSSAAIAAVLSVACDKMQNN

LHQELRQGYFGIPTGMQPQQGMFAQPGMFPMNAMMSPFVAMNTVSGAPTPPPMTNPQQFLNYANFFSNYWPQ

FANAANANAVNVMFQQQQQQQQQQQQQQHKQRAGGETK

>*Ostreococcus* tauri|Ot08g01530 New Gene model Clathrin-associated protein medium chain 6489: 7916 MW: 48257.875 Patched from Ol08g01410

MGVDSLFVLNNRTESLVAVKHWGAITSSEICERVFEARRDSARNGVEGDACVADQDSYGFHISRGEITYAATCSRETSPLLMIEFLSQLYDVLRAYFGDSVTEAVLQEHHVTLYQLLDEMVDSGVPVNMHAGGLKVLVPPPNLYNRVTSTVMGNQGIIVSDQDPLKLLPLPWRSNNIKYASNEIYLDLIESIDATIDAEGKVLSSAVYGSIEVNSRLSGMPDINLTLSNSHLIDEYNFHPSVRVSRFASDRVSFRPADGKSVLMSYKTANSDNPSSVPLPLYIRPQSAFGASHGRVSVVVGSKPAFEKPVESVSLDVRLPSRVLSADPSATHGEATFDVASNTVRWSIPKFPPDKTPCLSVQVNMRDEEEEATPSAGSKSDGASRRVHLQEVVDITASFKVPGAGVSGIKVETLQVRNEKYKPTQGVRYHTKSGAVVVRT

>*Ostreococcus* tauri|Ot09g03910 New Gene model Ubiquitin C-terminal hydrolase UCHL1 PEP - 591196: 592154 MW: 31038.566

MPKKWLPLEANPDVMNAFAHELGLSPSLAFHDVYGFDDDLLEFIPEPCVAVLMLFPLTPRTESVAGVDAPAPDAVSSVWFARQTVSNACGTMGVIHAALNAKDAVVPGSRLESLRAACEGSDPDARARVIENDDALEAAHVCASTEGQSAVPNADEVIDLHFVALVERDGGVWELDGRKPAPVYHGATTGSGLLRDAVPVIRKYMEAAEGSIHFNAIALAAKVENTCRYGEMRRARDVEATGVRASATRGRWMPHRVGVFRSGGAPRQMMERDIMQTPMGARWMSWC

>*Ostreococcus* tauri|Ot18g01040 14-3-3 9384: New Gene model 10456 MW: 29085.895 [Ostreococcus tauri]

MSGDGDDDARALSREDLVYKAKLAEQAERYEEMMDCMSEVTLKSGEEELSVEERNLLSVAFKNVIGARRASWRIVSSIETKEEAKHGKDSDKCKLIASYKANVEKELTSICTRILDLLKNHLEDRCSAGESKVFYKKMKGDYWRYLAEFKGGAERKEAAEETLLAYKSAEQTAEAELQSTHPIRLGLALNFSVFYYEILNAPERACQMAKKAFDEAISELDTLGEESYKDSTLIMQLLRDNLTLWTSDMNDSQATGGAEEEDDE

>*Ostreococcus* tauri|STE|MAP3K |Ot13g01170 New Gene model MAP3K 2869: 4821 MW: 69682.43 [Ostreococcus tauri]

MSCFRPATTVNEDGETSETTCASSFVAMMSRARRAIVGRGGGSAEVARNASAQSASGRDAQTTSAETNASGGGGEGASIDGEISAPRLPGLRLTPAGRVLAAAAAAAASGRDRGPDAEMNDEFGGMRGDPEVEETPLPLPPPLGDIPRAHGGVVGKIKGQILPSSGYVPLEVQTHAEKVRLIQQQERTRRSHSITDAAVTTTAKTAGAAVGAISKMAAKLKESRHFKSASAPLTAVEIADDAPAARESHAEEAGEANARSAHTEPTSNPFVQDESRRPGRNSSELSDSGVPSSPHHRRASSAGNWSDMSYTTSDDGMLRMCHTSLAPTKPRRWTKGDNLGEGSFGSVWLALNGDTGELFALKEVRFGSSDKHREESIEQLEQEVDVLSRLVHPNIVRYIGVTREEAALYIFLEYVPGGSIASLVHRFGKFEENVIRVYTRQLLIGLSYLHSQRVLHRDIKGANILVEKSGRIKLADFGMAKVLENVSHGKSFKGSACWMAPEVIRQKNVGFEADIWSVGCTVYEMATGAPPWSDCSTQVQIIFKIASSEEIPVIPEHLSPDGQDFLRLCLQRDATRRPEAVALLDEPFVVDAHRSSNATNIPGLQYDTWASSSVLSFRDDEASVRSGDGTVGGMRSNQSSMRHVAWGDLE

>*Ostreococcus* tauri|TKL|Ot11g00940 New Gene model TKL CTR1-like 169582: 172309 MW: 100334.7 [Ostreococcus tauri]

MFSRSNSDDVVEARAHATANAPDRDRIVGVKHEAASTRDSFVREVRDGENGSRATDDDASTSGDARDGGGEGSEEAGNVNGRSGRERGQSEGANAGRMALNARRPSRDHERTPNKFLPWLSNTPTLDGTLEKRQETEGWWASLFGGGTEQWGLRYFLLYDTHLFWGKGFSTMHGYGTVLSVREVPELGPTSFAVEMMAVPKRNLRRSFNDNVNFLDLISGLCCKPAGFRVMYLRATSPKEMQRWMEGFCRGTSDTPVSTRMPLDFSEEIPPSPRISTDSELSIDTFSGTGTRTRYEEPPPPLSPGGASREFLNTSPETEDVPLFRLDANGESGEFNHSTQQKSPVRTRSVLKSNSMLRNHEQRQSVDGNCSKSVTFDDHVESVVIARSPVKQQGIVPAQETDREFRTRTRLLKAAGSFKISESELQIGAKLGIGSFGVVYRAKWNDTDVAYKVMLQDKMNYETVNAFAEEIRMMRGLRHPNIVLFIGAVIQPNRLGIVSELMKRGNLEFLLHGNSTMGRQLRENGMLRRQMAADCARGMLYLHSLSRPVVHHDLKPANLVVDSNWTLKVSDFGMAQLKSYTYDSVSGAPGGTPEWMSPEALRGDKANERSDVYSFGVILWELMTVSFPWAELSSPVQIVAQVAFLHRRLKVPEWIEKPMADLLHSCWAREPEERPTFEKIVEQLAGEYPQAWSLGQVDKSADEQAANILAMMSTGKGASSTEADSADSSNEEEFVDNVVEVSVISAFAPRGLKPIRTPTPVTDVPKQTDTLDTEEDSSEESNSLDAPNGRASASANGTVMTTVNEFRPRLLRSRRRAPSSRPRSPPSQTAPAIDAVRCIITSHPSRALKIALGAVTTNWSTPAVDARPPARTARRSAQKPPAPLSTRTASRARASPARSWIPRCSRPR

>*Ostreococcus* tauri|Ot12g02970 New Gene model Histone H3 (Lys4) methyltransferase complex, subunit CPS25/DPY-30 (ISS) - 470097: 470484 MW: 12800.328 [Ostreococcus tauri]

MDDARCARSRARSIPRRDGTSDGRDVRGAAIGFLKRARRAEDAPTPSVPREDEPKAAEPSAEDSNERGESVKAYLEETVSKTLHDGMMRLANERPSAPLKFLGEYLLEKSRERGE

# Sequences for proteins with patched gene models

The following sequences are gene models that have been patched from *O. lucimarinus*. Amino acid sequence was transferred from the most similar *O. lucimarinus* gene to gaps in *O. tauri* where there was a high degree of conservation in the surrounding region. These sequences were used in phylogenetic analysis throughout this paper. Amino acid sequences that are derived from *O. lucimarinus* have been underlined.

>*Ostreococcus tauri*|Ot10g02860+A4S411

MDGERRARDDDDARDARSGGTRASASADEDGTNKSFDFAGCSQSQRDERRGATRGTARSQGNGESQEFGGPDFITPADAQFDAYGGYEDKENFRGARSPCALSPARNKRPRFGLDLGASQGTQEFASQPLDATQPSQSQGEFGGGFRVPRNREPTRGVGSRSGLPRAATSPPCARNAFLPDDEQPPETSAHARRANCTSAAQLATMSRFRADFVDLGCIARGGFSKVHKVIGRLDGCRYALKRTSEQLKNERDRSEALREVHVMASLVTCPQIVRYHSAWWENDHLYIHMELCEEGCASRMVDSRDGERMNDAQLARCLRDISTALAFAHDRGLAHMDVKPDNIFICKEGFKLGDWGRATQLSGARRSSAVDEGDARYLPPELLNDNFDNLDRSDVFSLGASLYELAIGTSLPSHGSDYQALRQGVVPTGAVAASLHALCVAMMSPIPLDRPSAADVLSKHCTL

>*Ostreococcus tauri*|Ot07g04140+A4S0Q4

MGAPRSPGGARASDDDARGRPSAPDELKNYEKLGRIGEGTYGVVYKARCRATNEIVALKRVRMDRERDGMPLTSLREIKILQRCAHENVISLKRVIQGETPSNVFLVFEYCEHEMARLIDFVKTKFTTSEVKSLMMQTLRAVEYLHERKVFHRDLKLSNLLLNQRGELKLCDFGLARTYDPIEAGIYTPKVVTLWYRAPELLFGEEQYTAAIDMWSCGCVFAEFLKHAPLFPASTEIELMQMICALLGNPNSNIWPGWDSLPHARKFKLPEQPYNFLEINFPKLSAAGVNLLDVLLTFDPEKRGTATEALAHPFFQESPPPKPPAEMPTYPSTHSAPERGAERRNAKRSRGALDERIGAVF

>*Ostreococcus tauri|CMGC|Ot01g05560+A4RRZ9*

MRKKERAAPAGDMFGEDSDEEAFYLRDASGEGEGGDGAKVTGGAVDMSKGLTDNWDDAEGYYCARIGEVLDGRYTITAHLGKGVFSNVLRAVDKREGEMEVAIKVIRCNQTMHKAAQLEIDILQKLSGSDPENKRHCVRFLRHFEYREHVFMVFESLSMNLREVIKKFGRNVGINIRAVQAYATQLFIALRHLKNCGVVHADIKPDNILVNETKSVLKVCDFGSAMFDGDNELTPYLVSRFYRAPEVILGLPYSHPMDLWSVGCCLYELFTGSIAFPGRSNNHMLKLMLELKGPVPQKVLRRALFTENHYDHTGAFAVIEEDPVTKKSIRRLIRDAKPTKDLTKIFTRDSDMSDGERK

*>Ostreococcus tauri*|CMGC|Ot07g04140+A4S411

MGAPRSPGGARASDDDARGRPSAPDELKNYEKLGRIGEGTYGVVYKARCRATNEIVALKRVRMDRERDGMPLTSLREIKILQRCAHENVISLKRVIQGETPSNVFLVFEYCEHEMARLIDFVKTKFTTSEVKSLMMQTLRAVEYLHERKVFHRDLKLSNLLLNQRGELKLCDFGLARTYDPIEAGIYTPKVVTLWYRAPELLFGEEQYTAAIDMWSCGCVFAEFLKHAPLFPASTEIELMQMICALLGNPNSNIWPGWDSLPHARKFKLPEQPYNFLEINFPKLSAAGVNLLDVLLTFDPEKRGTATEALAHPFFQESPPPKPPAEMPTYPSTHSAPERGAERRNAKRSRGALDERIGAVF

*>Ostreococcus tauri*|CMGC|Ot09g02480+A4S2P7

MTRSSSDTSSSDARDVDDVDSRSASSSDEDEGTDGYKRGGYHPVSIGERYNDDRYVVVKKLGWGHFSTCWLVEDVGSGREMNGKVTYRALKIQKSSGSYTEAAKDEIEILTQCKDQAASAEERELGSDNVVRLHDHFTHQGPNGTHVCMVFDVLGDNLLTLIKRYEYLGVPLLGVKALTRAMLRGLRYLHDVKNIIHTDLKPENVLLTLALPEKKRGRKSKNKVVDPRKDVKAGTPTLVDQIERLDVASSKRESGQVENDTSKETNSEDDIDCVDLLPYSLLKRLDAKICDLGNACWVDRQFTQDIQTRQYRAPEVILGAKYDTSADIWSLACIVFELATGDVLFDPRSGKDYDRDEDHLALMMELVGRMPKHLALSGKYSKEFFNRSGELRHIRSLKFWPCERVLIEKYNMPEKDAKDLSDFLVPMLDFNPSKRATAEKMLEHRWLQF

*>Ostreococcus tauri*|CMGC|Ot09g00650+A4S284

MRALGVGLIHPKTVQSFCEQLLVAVRYVHSLGLVHTDLKPENVLLMSNSYRENATHRVPVDHTIRLIDFGSTTFIDRHHSAVVSTRHYRAPEIILGLGWSYPCDMWSIGCMMIELLTGEALFQTHDNLEHLAMMQHALSRTIPNAVVKRVPKDKLRDLFNRNGALNWPNEKTDAESYAALGNTGVVRQLLEKHLSGEVLSLFADLVGKLLDFDPKRRITSKSAVNHAFFSLDLKIDWRIMRNGDGRVLGVKK

*>Ostreococcus tauri*|Ot15g02970+A4S7Z1

DYDNRTPLHVAASEGSFAVADWLVKSGVTINPVDRWGSTPLESAVYGNHSDLVKMLAKNGAKIKDRVSGTFVPLEESHLSGVFHTQLPADTMAWEIPDGEFRTSKRLVPALGVVNTGMWPGTKVCLKQLHKHLNADEVAQAEFRLELKIMQQLHHPHIVQFLGTTTSTEGLTSIVSEFMGGGSLEQVFRNEELLSLKLATQMALDCARGMAYLHGRSPLPVIHRDLKPGNLMLTTNRTLKIGDFGLSKTLSVRNKLPQEMSQAFNMTGETGSYRYMAPEVFRHEFYGPAV

DVYAASMIYYQLFSFQQPFSGRNPVDACRAARLHAPRSRRLVTRMWDPIVKKRPDFTE

IIQILTPVAARYAAQPDASAAGPACCVVQ

>Ostreococcus tauri|Ot04g05350+A4RWS0

MRSSSTSRRAANDLARGTALGTHGRYEIVEMINRGGLDRVSRHQLHANLHPAALTTDALVLMTTEMVGLYFLSTVLLVREQLPEEYRAIITEALGADLEFRFYAKFYELIFMASAALTVISLYAKHVTAASTVKVPLAARRAQIKYATRLMGGNGSNAGVPGVVRLLNVVNHGEDVLVLVFELAKGVDALDYINQHGGRLDEDEARRLFAQLVEAVRAIHDLGFCHRDVKPENAIVCEDGNLRLIDFGLAKGLESAKTRAVGTPDYMSPELLDKLDRDGKARTEKYDA

AACDVWSSGVFLFIMLTGRYPFQDPNRPNNVKATLQNIVKGNVAPLRVDVSDDVRDLLRCMLTPDPRKRITLKQVAKHPWLSGSLGRRKKPSLVDRLKQSFTFRRATTSD

*>Ostreococcus tauri*|AGC|Ot09g00870+[A4S2A2](http://www.uniprot.org/uniprot/A4S2A2)

MCVEDFEPLKLIGRGAFGEVRLVRQKDTGEIFAMKKLKKSEMVRRGQVDHVKAERNLLAEVNSRAVVKLYYSFQDDQFLYLVMEFLPGGDVMTLLIRRDTLTEEETRFYLAQTVAALETIHRHNFIHRDIKPDNLLLDKDGHMKLSDFGLCKPIDPTYQQAVLPEILENESMGPSDPPPRTDEDSKRAEEVANWQKSRRRLAFSTVGTPDYIAPEVLLKKGYGLECDWWSVGAIAFEMLMGYAPFYSDEPLTTCRKIVHWRHHLRIPDDSPISPEARDLIERLLCDADQRLGTHGGVEEIKSHKFFKGLDWNALYTMTPPYRPVVSHRLDTRNFEDFEEDDSMRKKSGTVEGEGAPKDENFIGYTYKNVEIVGEEVKKLNIARPSLTSVFPTSK

*>Ostreococcus tauri*|MAP3K|STE|Ot11g01380+A4S4M0 (NB incorrect n terminal sequence removed before genomic gap)

MGRTKQRGKTLTLGEFFAETSTQRTSGLLGARLARAVDGATDGGDDGDDDSYAEFTVEIGNASVRCAEQEGSYDVRAFAREGRNAGGKVIKSVTFVFGGKMGERRVTEAPFEVQYRCETSVDVEVAVQFHRALNARPVRELHAIELSSEEREFSRSFAVEVKRRALMKVLGKDKGDAEVDVRRAESRSSLLEESQSRGVDAWTVQDVSEWLRSIELEELVERFAKAKINGYELLRLTEKDLRESLHLERNLERVRAIRAINVLRASAGAADAENDDKSATPPLSAPLGAPRGGLSPLELELDVSWIEFVSEKARASVLIGWFMHVLDEVKAKEFDEPGPQLSVYCEATLQASKSCEDALEERVLDILESTPGWDPRTKMFPSTCDLTKLNDQLMELYLEVKAFEEFAALNMDDDRGEHERSRSTPPKMPGESSPAIPRLNRAESLTTPSVSGSPTFTSPRNDMGPSPSPSGLNVGARSFSPTPLSDLTRSSLPELQEDEVADGDRVMELNTEWEIDYNDIEFEGGVPSSKNRIGHGGFGEVFLGRYHGSLVAVKKLFNQDMMGKGLQDFRREVRILSRLRHPSIVLWLGACTQAPNLTIVLEYMDKGSLHQFLHRTTTPYTTLTLTRWAMTIAQGMVYLHSAKPFPIVHCDLNTNNVLVNRDGMVKITDFGLSKVKHSSRLSRQTGMTGTVNYASPEVIRGGKFSEASDVFAYGVILWELLTRRIPWEDLNEYQIVFQMTSDLDASLAATAKNLELPASAPEGYRKIIHGAWATQPERRSAFKDVLGDLREVYREQVDIEKALRAARKGSTSSLSATSAPDK

*>Ostreococcus tauri*|MAP2K|STE|Ot04g04050+A4RWE8

ECGERDGFISECGERDGFILGAADLELVGVIGTGSGGVVRLATHKRTGEALAVKTIAISLARDENERKRIVTELRTLHKSECDYIVRSSGAYFDRGSVSLVMEYMDGGTMSDATKYLGKWVEQDLAAATSMLADGLHYLHTKLNVVHRDIKPCNVLLNLRGEAKLSDFGVSGHLTDASKCHSWVGTVTYMSPERIQGESYEYTADVWSFALTMVECALGRFPYNPPDVSRRLVFWDLLDIVVQDPVPNLRPELDVSDEFDNFVALGLNKDPTGRMLTKNMIAHPWIFGRDRASDKRRIAALAARHLDARSKARERGEK

*>Ostreococcus tauri*|ABC1|Ot08g00750+A4S118 (second gap patched)

MPLTSRPLQTRENQPSNRIRSSRAPFQRLNHTIHRPRSLVRPIHLAQPHVHRVLLRLSIRIAPLSSSTNRRRRRRARVSSVQRPRRSALSHEHSLDRSHRSPARRPRPPRLFLPLRALTPVHALRERRHHTNQGRHVPEQRIXXXXXXXVGRRTTGDADDDDGDGKRDARRRTGRGVHRRARRAHRSADPSRRSWNRFGLARLRPRRANRRLRKGLATTRERGTALAISGPPVIGKSLEEPIEAFNKVGFVGKLRFGLRAVGGAAKAFGVAAASATLRCWAPKVHGALTVMLVACASTKRGGDGALVRFVEGKVPGKLGRACVRAMAAAPKSARGTLRRWATWQAIRLGMRLTARRQQRKVDYSMRVAPVIASYMIAKRRIGRIKEVERRDVEWEKQHHWGAARMRDVIEDFGGFYRKVGQIAGTAKQMMPAPYIECFSKTMDNNPPVSFRLVRRTLEEGLGGPLGLHFAELSRKPVATASIAQVHFGRLLDGREVAVKVQATDAAMMIGDIESMLSTTRAMRWLGLDEGLDFPTIFRAYLDVIEEEFDFTIEASKMDEFRKVLDAAGLGDRLAVPQVITATRRVLIMRRVRGVKLLTLFNRARATNKIPRCPSPVAQCHAVGGFGWQGVFHSMFMAWGTMMLKHGHFHTDPHPGNFMVAHDGKLILLDWGQTKRVSEVERMHMCRLSLYMANEDHTNIAKEIRSHGSVRLERPTTEALSALS

YAYFDTRPSPLAEMNVMDFKNSPFVQNKILQNTQEGFFAIRSVFLLRGMLSTCALRMSMVQAWEPIARSAMILNGEAPPSRFRLKTQFIINRSLLGFQRRFNVGAGARVNAVDEYMSTKRDKDEANGTLWPNSSSLF

*>Ostreococcus tauri*|ABC1|Ot03g01200+A4RU11

XPDALAPWRGELRYDVEAFEAYFKRRPMKVLRRALVVAYELGAIGLGVAARAGERRRRAQRLSETLARLGPAYIKLGQVMSTRADVFPVEYVHAATSLETLERELGLGVDFFEKFHETPVAAASLAQVYRATLPGGQDVAIKLQRPGLAELVALDAVILRRFAGFVGYWRNFKSDVVGIVDELVGRIFEEMDYNKEAESCERFRAMYAPDGDAGVGLAGLVYAPRVVDFLSTATVLTMEGIVPNEVLDRGLRASSSAADVGIMHTDPHPGNLIVAENGGLTYLDFGMTVEVPIETRRAMVRGLIGFVNRDARGLVDDLKVMDFLPPNVDRAAAEEALRSVFAGESTTKVRNSNDFMGVVSQLSTALMKHGFRLPPYFSRILRALAALEGTATTIDPSFRVVDRSYPFVLSRVLSDRSPEMRESLRRLLLAEDGSIRYKRLIRLIRAYGVEATPMASSETETRSDCRQALDKTIAGLSELASGEKESLSGEDEARRATKTAMEDALRFLLSDRGESTRERLIDDFIAAFEALLDEDSGSKTDIPEGRFTVDDAIGAAKSAATAVGDNPDLWIPVLGRAASERETLAALRASSRVAYDRLIKLRASRRSLGRAPCEDAKELVRKVVHALSAPKSPETQPTRDSSV

*>Ostreococcus tauri*|AGP-like|Ot06g01800+A4RYP1

MTRTTDGTRGVADASREGWTTRARGASGRRRTVGERWALDDATLGRGSFATVWRATCVKTGAVVAVKEIACERLSKKLRESLKLEVEVMRRMRDENILRFIDMQSSNETVYIVLEYCAGGDLSQFIRRNGRMNETSARRFMLQLARGLKAMRKAQLVHRDLKPQNLLLTSNDLNAELKIADFGFARYIRDSEGMADTVCGSPLYMAPEVLNYQKYDAKADLWSVGAILFEMLVGTVPFTGQNQVQLLRNIQKTEFKIPIHIAEDLSPACIDLLRGLLHRNANDRISFEDFFNHPFLKSGDTVGVGIPSKSGATKSQAQESDGASSADSETMPFNMDVESNSPSPTSTTRVNGQPQERTIS

QPVPMLSRQSSASGKMSIASDYVLVSSPGTSIPRSMRPPSLGSSPLSRMSLSPNDGSPGTFGGRARMSRGTSPSSQPMTLATRYQTQSQMLVTQLGVRVGVLEKAAVVLRDTSTEHWNVGNKLAALSLGLVSLAALRSAHRLASEIVTTEQKSSVNLSGTSASASSGNSSPTGGNSMAMSAKKATVRIKEAYQAAHTRAEKAADACRAAGFDMEAQLPDGMELVYENVCKLAKDGVREELAENKQVALDIYGRAQTLLQFLIGEGPSLHITPPLVIDGTTHARLSSLSATVASRQQSLVRLMRGVR

*>Ostreococcus tauri*|Ot08g01230+XP_001419308.1

MGLSTYEQQAERLMSKSATTSMRLEVATSVRDGIEIVHTSEYVNFLKSCFPAFKEILTKHTSPTMEDNEMNALRHVVYEILNRLPFNEVLRPYESELLDLALESLREENEKNALLCLRVIFDLHRNFRPTMDKRVEPFIRFVHDVYEGSGVTIDELLGSSGSTRKRGKSEEKLPAPCELPSHKSFKVMMECPLIVMLVYQLYNRDKFIQQEVEKMIPLMVKFTGLQTIDIDSMSAGQREFSSDLKSAQVKTIGFITFLLRGNSVFVEPHHEEISNAIVSLLRTCPDVVATRKELLIATRHVLSSPPLCKGFFQHLDLMMDEDVLVGTGRMCIENLRPLAYSFLAELVHHMKAELTLEQIRRAIHIFSRNMQDETLPLSTHMTCVRLMHHLVESVFRMRTDASRATEAREFLVRIMYATVAKFRTLRPNIPELLKTASGLEAAVKAKASKRSKDAKADGVAETGATETPNKSASEKKESSDDPEPDLPSHEKLRSLSDNKAIIKTLTIGMKTLLWSITNFNGSESVDIGLNRSEIKRASGFIRNGVKCMQLFNGTECTEMCTHFSEALLVLEPRNFVDMMSLHFDDFFAGMLELPTMVQVPHLLLQNTKLCRYFADAAMTYLLEKKLECLRDQSSQEAQLVLKLFSLLLHAISKHSHCESVLSTHVIFMMDACLKAIRENDNPSAYVRLLRYLFRAMAQAKFDVLYQEVVPILPATLDCLLAMLNGPDPLELHDTVVELCLILPARLSSILPHLPKLAMPLCRALRAHANELNLLGLRTLEFWVDSLNPEFLDPCIAEVESELMLALWAMLKPQQSGSPFGAKAMQLLGKLGGRNRAFLKNPLKLDAKSNPEHGLRMILMFKPDTSFLVPLDRCIPSEALVRHRRQALTFIRTCLVSILNLSAARALYGTVEEIESALEAAISNGWGKKCEAMTGVEATPQLGNKTKGQLVAEQNIFKRLVVTVIAAECDLSLKETDEKFMDNVCEHVALLFVNSATCTPGEDTDNMDHDSVERPRATNLKVLETTLFLDALMTSFESTKQIYLTATVKALATFLDAVLILSRDELQSLQTADDSLEKQRTPKSKSKKKTDIHDDESPYPILASLVEAVLPRFVHCCYKREPHSIIGGVEGFNLLIERLPTSIWRRRLPDILSANIRAIQLLPAHAPAQKKRVEQVFLQLVEKFIPAEVSPRGDDAPTGVDAGVSVLVDELCNLSSTCASRAAIERALLGISERTSTPMDTVLSITDEKLMTVFERPLLTRHVLSQIQTVKLINFCLNASPHLITFRDKWENNLRAFINEALTIAEVEDPTIMSVETRENEAMSTLRQSCVQLISSALKAPEFSDADTAEELTVIRERISTVLFTSLTSRNKVIVEIAKKGLVEVKPYMNKNTLKQSVSPILMNLQHLSKLSVPLLEALEYLLELLAEWFSPTLGEKLLEHIKYWIELDFSAPAVPGQLRKSPKDAKLIAAIVNLFHLLPKLRDKNELKPNEKRLLPFDFIKPLVRDIMVIESTMPPASVYSSAHSLFIVPLTKFLNRYPKESVAYFLARLDQPEHFARFVSILKLPEAADLLKTITSSSSKLLHIIFGAGKGEMDEIDKIAVEGVEPIEDTKLAYYNGLQLLATIAKLKPDWLPKEKAILKQLNECWDSNDRAATLSDESKVSLPAMMETKYLAKCFLSIVKNDHSQVEILFKIISVLCTRSSVDFTFVREFVKNEVVERYTPAERNAVLKQFLKEFAEQLEAIEDANPDHVTCSLKVMVNPMLEKSLTDGRDDDGEPSDKLLEIVTDDFVSELVTDVFEPSDGGNDALYTESVLIQLLQLSTLLIRYVPKTLVDHRKELIKFGWNHLKREDSSSKQWAFVNISYFLQHVVALLRAYQPEQKALVTEPSKKAKKEECEAKSAGRSTRASRSSKAPKDDDDDDTADEMMSDLPETETLNSMPSIGVIDEGFKPSPAMEEIIVNFLVRMTFITCEGKEQDSKELNTRTVELLSTALEMWPSARIKFTFVDKLLTLANQSNRDPTSTLLMSLSVLRRAMKISDYKFFSENVEQTLSLIEPCLSSTSERAHSLLAEVVGMAFDRFKSKDNTEPESCEIQLRHQLDRVYTRAIADATVSKSLPNAEHASTTLACTLKVMATEGALRTAMIDENLPKLMKVLARLTHEFNQASAAGDIQPIHPKRGQTPPEVVQPEYGSVANCMVMCIDIISKRVINAGSEQKQIFLRLLLQLINDKSTHGNVLMAILDAMIAWADDSTLGEAVSDDERNSRVGSLNAKETVLFLSKLAQLTRMGLAITQTQEWEEKLLNVTYKLCSAEGKHEPALRSEVFLKVERLHLLGLRTRRPELRKKFFSLYHEVIGKSLFQRLQYILCIQDWDAMADTFWLMQGLDLILSTLAEDERIMLAPNSALISPLLPIDLETKNPLPVPSKPKNAKSNSPELDELIKRHAKFLHEKSNISVNDLMTPLRQVATRNAHIAYYLWVLIFPIVWATLQREEQLQLAKPMIGLLSKESHLRQAAVRPNVIQALLEGISLSQPQLKIPSELTKFLGKTFNAWHTAIALLENHVVRYPQEARCFDALSELYRLLNEQDVLAGLWMQRCHSDVTRAGLSLSQHGHWQNAQEVFFEGIQLATAGQAPGVSKTEMCLWETQWLNSAMQLNQWDLISDFSRTVEHSELMVQSMWRLSDWAGVKELLPSGALNETEETPEITTVRAFASLVSGRVREAEQHWANAVKSSLDRWWRLPETGSTCHIPSLHVFNAIAEVQESTRILLELSNTQRRGAQGLANNRTLVQDIMETWRLRTPNEWDQMPWWNEILMWRGNMHNIMTHAAKQIGEQNPAMLQVTQQLDQLGQRERAWSLNKFANAARKQNLPEVALNILNRHQGQIEVSEAFSKLREQCESYLSLGDEAVTGLNLLESQSLEFFAPPQKAELFRLRAKFQEQMEDYSGAYTSYATAVTLCKQLAEGWISWGHFLRKHRNEGTGLMQATTCLLQGVRNNVQENRHELLHVVRMLAFDANTSAVGGAIMRHLEYLPKWVWIPWIPQLLLSLGHNETQYARSILLQIVAAYPQALYYQLRTSLLERRDAAARATQTARKLAAKDDEKGKSEAKASYADIQAAAQRAQEATIAFEAAKEVMEQLRVRNTNLVGELEVLLSELGTQFACTPEERLLVVVCTLLHRCYKYPAATTGEVPENFKKELIGVYQACFSADTSVKHADFVKEYKASYERDLNPEQKTFPKTVAELMSKLKGWKQRLMNDVEDSLPASLRLEDESMALRHVTFNEIEVPGQYANISYGVTDRFMKLNRIGADVHIVRRNGNCFRRLEFLGTDGSIKQFIVQTSLTPAARGEERMLQFLTNLNDVFAKHAETRRRNMCYYTPAIIPVWPQVRLLEDDDNHGTYQEVYDANFARYGREADLPITLFKAALDPAILGEVTGAEDVLELRLKALMEITQKHVTENIFSQYMYKTLPNSSHLWTFKRQLSQQLAMSSFLSALLRIGGRTPNKIMFAKNTGKIFMLDFHPAFDQKGIVEYIEPVPFRLTRNLHTFFTPFGVKGDFVASMASAAQACTAPEADLEAHLELFFRDQLMVWPWRRMGGDTPPVSPSPSEIKNMARANVQEVLRRLPIIAPTPVLPDTSDTVPSVQKAVLHLVDAALNIRNVSRSAAIWAPWY

>*Ostreococcus tauri*|Phosphatase_2C|Ot03g04580+A4RUZ8

MRASVRACAGSGALDAVSDGENAAERNFGDGETGTATGDLRGRTYVVTGTETSAIAAACARRLRDAGARVVCACVDANEARARVDGDKGDGRDEDATTANTAHERELRASGALVTMYCDVERLETIERFAREFCAKAWALDGVLNAASTTMEEFSLTADGIERQFAVNHLAHFKLTALLMDELVRTAAASGREGRVVYLTSNLHHFSFRIRQGTPKPSRGIDFANINSDFGYSPLNAYGQSKLANVLHAWSLSERLAKSGARVRCVAATPGLTELELDRSLSFPGGSLISAPLKYVMINTLEEAVVTPLYCLTAPTIPPGTYFNNCVPVKASLPGRDPRLAARLWEFSEELCDAGASSVRATARSLPAYLYYRLTRGGRTHDPGYIEQLSGTRAERLTNFIARTQVEKAAPWWIWIVIACATIGISFAGVLLQNELYETPPITRACWRLTLTTFFLAPMGAWEYRRWESSAERDKMRAGKTWGILLGSGFALGVHFAAWVASLDMTSLAHSLLFVTTSPLLILVENAIFRRHRPTNMETAGVFIGLVGAGITLLDIRDDKEVTAKGDALAFLGAVAIVFHIECGRTLRTWMPTTVYAFPVTLIAAVFLALFALVFDEREPVFGWASSAKISWFVLLAFVSGIIGHAGFNFALGYVSSLVVSISTTMEPVIGTIIGFLTYGTSTPKLFTLLGGPLLLAGIRADAGGAASVVRALGDACDGEASGTTVVLALVRSDGFEVAHVGDSAAYACETSGGGVRARRLTEDHGLGNARERARVRAEGGTIVESRGAMRVGGEYLVTRALGGTARRARGVSQTPDVVARRWRTNELGLILTSDGTTERLKPDDACAFVFAGKRKCDESAKVDTSIALDGDAASSTQSSEDAWDTVEVQGNSEVMNERVERALKCALELGSSDNVALAAVSAGRHNTQITVTQQKPSSLVAEMLSIGSNIEEYKITQLVAWSSGPYLPAKTPFRYDDMYDEEPQPTYNFGRKFEQSAIEGALTALALAPGKGEKNRVQNAITSYVGIIDSRLARGQDEGVRAGERPFARGHFGEVWRAKLSRQLTAQKLECASSAVSGDSSTVIMKRILVEQDLDLQLSADREVYFGRLLCGASPHIARYMHTFDKIPPSGRERWLVFRDEGESLERLMYAPESEVGGDSATLQLVSQSDWWRETRRSATGRRVLKTILRQIFAAVNVSHSNFGVVHRDIKPANVFVRFDDDIVQAKLGDFGSAMDTRRRIQLYGSTGPSAAQETAEYSPPEVLFGNDLIERTLKYDIWSLGVMMTELLSLGSPKAFSHISRKTRLALERELREVHPTARAVAYRLRAMLELCIVPPDTQVGTLLSWECTEVALMNIFKARDPLGVGLESIWALRLIRKLLSWDPNERPSAAQALEHAFFRDGDERGWKCGADASEHEWKSRCDALCASPCT

>*Ostreococcus tauri*|Phosphatase_2C|Ot09g03800+A4S313

MGAYLSQPVTRKDSTDGADARFAYGTTAMQGWRTNARDAHATIPELDGRTAFFAVFDGHGGKEVALYAARHLHESLKETEGFEGDGDALKGALEESFLALDRRMLSKEAASELRALRSGSGEEEDGALGGLVRTGANAEEQRNRRAEINAKLRAALIEQMKEQDPNIDERDIKFDFELEDSDFDEPQPSSSEDGGVDALDRWSGPQAGATSVVVCIRGNNAYCANAGDSRAVFSRKGGVAEDMSNDHKPMNEEERKRIMNAGGFVSEGRVNGSLALSRALGDFEYKRNKDLSEKEQAVTAFPEIREFELREGDEFMILACDGIWDVMSSQECVTFVRERLIAGAKSETFKISRVCEELCDACLAPDTRGSGLGCDNMSVVIVLLQKFWQPTSA

>*Ostreococcus tauri*|Phosphatase_2C|Ot01g02430+Q01GJ0

MRVNAWRSRGRGGGGVGTTRGGARRARGGTAARAAATRLEVSSAGAILVPHPDKADKGGEDACFVLKQSGAFGVFDGVGGWAEEWVDPAEYSEKFAEKSAQSVLRGTRDPVAVMKDAHDETQVIGSCTACIAMLKDGNILDVANLGDAGALVAREGEVVYQTSPQQHEFNLPYQLGWAKVYPEGDRPEASERSEISLSPGDVLVLGSDGLWDNVPHAEVAALCAEHNGDAEECAEAIATLAFGYSCDPEYDSPFTQQARAVAETRPEWGDRRSIIGGKMDDIAVVVAFIDSERTLS

>*Ostreococcus tauri*|PP|Ot10g01220+A4S3K6

MLGATFARDGRARGFDDATNARFTTNQRHDGDRAKTTRDDASPGARCGHTLTALRWNQRTKIVCFGGATELEGASASALGDGRGGSPLHGGGRDGTNWVKLSGATSDLRVFDPQGGEWDELKSGGDVPSARAAHGAPTVGGMLVVHGGIGPDGLARRRLVRVGSGDEGSERYAHVLSFVAQRFLVVIGGTDGSKCLGDTWVLDTTTKPYAWSKCNPTGPTPSPRTYASASTRTDGLLLLCGGRGADGMALNDAFGLARHRDGRWEWAEAPGKAPTRRFQHATAFVDTRLHITGGASAGGQLVPEETTMSMLDTSAGGSTGWRECKRDGPKTGLVQDANALVGPRCRHASVSYGPFIFVHGGLRNGTLEEGELTPCAALRHRLSSSLGGAWGTGSGAPPSFRVARVNEDSKHAHNELVSCVSSRGDDYITGGWDGTLRTWRWDPTKGLSGGAPMTGQHNDNVEFLSVDVREDHEHLAISGGRDCTVRIWDVAKRSQRGRIYAFENIASGCVDWPSQTVAVGSRGGAVMLWDAEKGTKKCTLRGHEGEITSMCTYDWNTRDTFAINTQRDATYSNGSFSSPSSPPEVTLRPLGGSPSSPDSPEGMNANAEQELRHASAQEAAAALDLVARRKFSLGGSDSGSPGGSSVHTPSPGFAGLGSPGDARRTPASEVRLHHRAVVVAAAPYDSESKSTPRGVASMVRQLSIDQFENEARRIGTPGADMYTPGDTPAKLARARRAAELGAQPVHRVVITHLLHPHTWEPSQDRRFFLNAAAINELCDAAEHCFKNEETVLRVNGPAKIFGDLHGQFGDLMRLFAEYGSPSTAGDIAYIDYVFLGDYVDRGAYSLETISLLLALKIEYPNSVHLLRGNHEESDINGLFGFRIECVERLGEAVGDQVWRRFNSLFEWLPLAAVIEDRICCMHGGIGRSVTHLSQINDLKRPLTMENGGVELMDILWSDPTENDGIEGLRPNARGPGLVTFGPDRVRAFCETNGIQMIVRAHECVMDGFERFAQGQLLTVFSATNYCGTANNAGAILVLGRDLTLYPKLIHPLPPIAMESLSPSDRIDDNLWLQDVNRERPPTPPRGRFGQSQPTIGLINPI

>*Ostreococcus tauri*|LST8|Ot06g02150+XP_001418442.1

MPTPSVVLATAGYDHTVRFWEATRGVCYRTLQYADSQVNRLEITPDKRYLAAAGNPHVRLYEVNASNPQPVTSYDGHTGNVTAVGFEPRGSWMYTGSEDGTVKIWDLRAGGYQREYESRGAVTSVVLHPNGTELMSADQNGNIRVWDLTANACSCELVPEVGTAVQSLTVAGDGSMVVAGNSKGTCYVWKLQPGSKTTAHFEPLHKLNSHNGYVLKCLISPDCRLLATTSSDKTVKLWNLDGFKLERVLEGHQRWVWDCVFSVDAAYLVTASSDTTARLWDCSTGEAIRVYSGHHKAVVCCALNDSAVSDAVEAETDINSP

>*Ostreococcus tauri*|RAPTOR|Ot06g01080+A4RYH6

MKISPCARMQCWIQPMTTQPQNELDAIGKALQAQYERWQPRAKYKLQLDPTTEDMKKLCISCRRNAKNERVLLHYNGHGVPRPTANGEIWVFNKSYTQYIPLSVYDLHSWTGTPAIYVFDCSNAGLIVKSFLKLSDPAQPQPKAGVDLEQRQSGGSMAFAQLEHGMRSGVQDSGFGPSATANSANECILIAACGANELLPQSSELPADIFSACLTTPVKMALHWFCSNSVLHEHGITVDIIDKIPGMQNNRKTPLGELNWIFTAITDTIAWNVLPRKLFQRLFRQDLLVASLFRNFLLAERVMRANNCTPISSPRLPPTHQHPMWAAWDMAVEQCLLQMPSLISGDPDVEFVPSPFFTDQLTAFEVWLEYGSEHDSPPEQLPIVLQVLLSQSHRLRALILLGRFLDLGLWAVELALSVGIFPYVLKLLQTTAPELRQILVFIWTKILALDRSCQADLVKDDSYNYFVRFLQSSSVPVEERAMAAFILAVICDGHEKGQSVCLASGLLNICLSNIHDAAQPETGSPFFLRWLCLCLGKLWEENFEAQKVACSAKAHEQLIPLFSHSSPDTRAAAVYACGTFISFGCVRESHQDDDIDRQESHEPLMHAMSSQNVLGTKTSGSGSADTPNTVGLFISPGAIASMEITIAYQLLRTAEDASPMVRIETAVALARIATAHSMMIRESLVAWKRDYDSVTAKRVVDAKRSEAMGVRRRFSSGTSFSGMDHSLGSNMSTVDEDDNMPMSVESVARSADEQYATSDNTMPTTAGTLPPPFPRSHDSADPVESRTPEGTKGTVYTLILQALLNLATDPVPAVAKAGCHALHAADIDQSHPIMQMVTKAGNFAEVEPVVRRLSASQRRRPSFASLDFGTHHERSFSKDVGVSPNGGGLTRTDSWHQRLANLGSSLGSRLLGSPTKSKPVSKLSGNRSEQQLSPSTGSVGLRTSTIRGKGRKSQALDSPVSSAGHLPALVIPSTRTEQLRNSPTSLSTLRRTTSNLITDHSVEQDSLGQQRIFGSHASLDSLDSTLSPRLNRSEAMHGYASDDVHDAQLPKSIIYKRSCGHFSTPLLETAQDEDDDDDDEIESSISPWMRPPDMKRRAERLKMIRERSKETLGGGERRLKMTEYVSSIDVGGGVTPTSILMHPFEPHAFTGDSTGRVHIWNHNSSRMVNKIETGMKSVNLLSLVNETDDALLLTGCEDGTVKIWRSYDSIQKETLVTAWNTLPAQPIVSENGEIRSGLPLSFYKPSTHYKAHESTQAAIVWQQLTGCLYATGNAPNPFLRVWDVSSEICRDTLNLQAQGTCLTAEGALLMAGTHDGAVLSYDLRAPARLLSAMQTHTTPVISILLQPGGVNNLLVTGCSEGQLKFCDLRNASKPFLITEVTHCARWSLACSSHRIWFIARVIASGSSERAIKLWDLRGHNFAAIQYTNSFLGQRIGSVTALAFHPNSAYLAAGSNAGHATIYGPHF
