# Supplementary material for: The reduced kinome of Ostreococcus tauri: core eukaryotic signalling components in a tractable model species
Source: BMC Genomics. 2014 Aug 2;15:640. doi: 10.1186/1471-2164-15-640 (PMC4143559; doi:10.1186/1471-2164-15-640)
Supplement: Supplementary file 4 — Additional file 4: Figure S3: Evidence for conservation of phospho-site regions in O tauri. Sequence alignments for conserved phospho-site regions implicated in the phosphorylation of (A) MAP2K (Ot04g04050) by MAP3K and GSK3, (B) S6K (Ot07g02590) by GSK3, (C) S6K (Ot07g02590) by TORC1, (D) S6K (Ot07g02590) by PDK1 and (E) CCA1 (Ot06g02530) by CK2. Black framed amino acids specify a conserved phosphorylation site, and red framed residues show the conservation of a pre-primed phosphorylation site. (DOCX 160 KB) [file 12864_2014_6366_MOESM4_ESM.docx]

Evidence for conserved phospho-sites

The following figures show supporting evidence for **(A)** MAP2K phosphorylation by MAP3K and GSK3. We also show conserved S6K phosphorylation sites for **(B)** GSK3, **(C)** TOR and **(D)** PDK1. **(E)** The conserved CK2 phosphorylation motif for CCA1 is also shown.

1. Conservation of the *O. tauri* MAP2K (Ot04g04050) residues for phosphorylation by MAP3K at S178 (orange) and GSK3 phosphorylation at S182 and T186 (black).


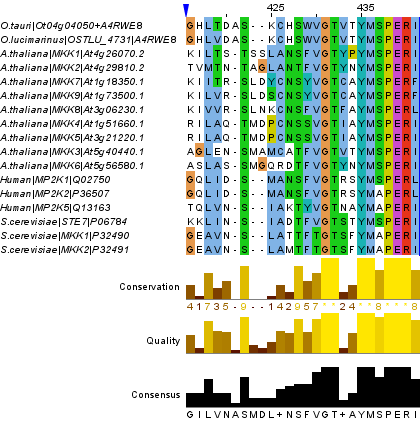


1. Conservation of residues for GSK3 mediated (S371) phosphorylation of S6K (Ot07g02590), which is part of the TORC1 pathway, in *A. thaliana* and *O. tauri*. The proposed GSK3 phosphorylation site is highlighted in black. The upstream conserved S/T residue which could act as the GSK3 primer site is highlighted in red. The GSK3 target site is not conserved in yeast.


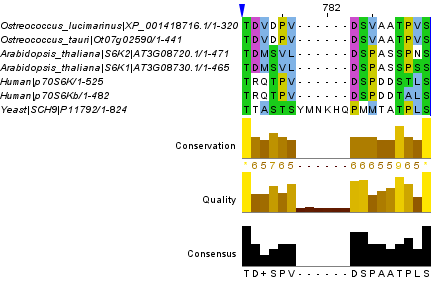


1. Conservation of the residues for TORC1 mediated (S389) phosphorylation of S6K (Ot07g02590), and subsequent PDK1 binding, in *A. thaliana* and *O. tauri*.


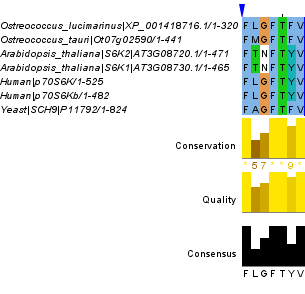


1. Conservation of the residues necessary for PDK1 mediated (T229) phosphorylation of S6K (Ot07g02590), in *A. thaliana* and *O. tauri*. The gene model for Ot07g02590 has been altered. The existing gene model has been corrected because of a gene fusion and frameshift in this activating-loop region (Supplemental Figure S2).


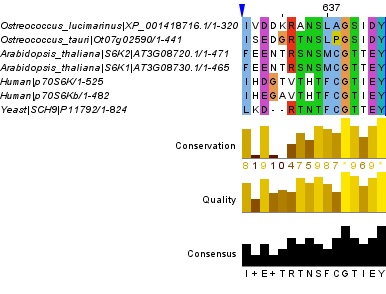


1. Conservation of a CK2 motif residues in CCA1 (Ot06g02530) (black). Upstream CK2 [T/S]-X-X-[D/E] motifs are also present in *O. tauri*.


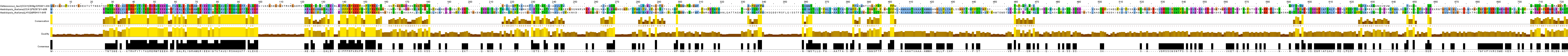
.
