# Supplementary material for: The reduced kinome of Ostreococcus tauri: core eukaryotic signalling components in a tractable model species
Source: BMC Genomics. 2014 Aug 2;15:640. doi: 10.1186/1471-2164-15-640 (PMC4143559; doi:10.1186/1471-2164-15-640)
Supplement: Supplementary file 6 — Additional file 6: Figure S4: Kinase similarities between O. tauri and other model organisms. Box and whiskers plot that describes for each species the distributions of the Blast Score Ratio (BSR) similarities of O. tauri protein kinases against the best hit in the given species. BSR scores for each O. tauri kinase are calculated against the best hit (highest score) found in H. sapiens, S. cerevisiae, A. thaliana, and O. lucimarinus. Red crosses show the outliers, whiskers indicate the extremes of the distribution (excluding outliers). Boxes show the upper and lower quartiles, dissected by the red median line. The blue dot indicates the mean. Notches, indicating the 95% confidence interval, were calculated from 100,000 bootstraps. Table one of Additional file 6 gives the significance of the differences between species for the mean Blast Score Ratio (BSR) similarities of kinases. (DOCX 64 KB) [file 12864_2014_6366_MOESM6_ESM.docx]

Kinase similarities between *O. tauri* and other model organisms

The following box and whiskers plot shows, for each species, the distributions of the Blast Score Ratio (BSR) similarities of *O. tauri* protein kinases against the best hit in the given species. BSR scores for each *O. tauri* kinase are calculated against the best hit (highest score) found in *H. sapiens*, *S. cerevisiae*, *A. thaliana*, and *O. lucimarinus*. Red crosses show the outliers, whiskers indicate the extremes of the distribution (excluding outliers). Boxes show the upper and lower quartiles, dissected by the red median line. The blue dot indicates the mean. Notches, indicating the 95% confidence interval, were calculated from 100,000 bootstraps. This data shows that *O. tauri* kinases share the largest (median) sequence similarity with *O. lucimarinus*, as expected, followed by *A. thaliana* and then *H. sapiens*. The furthest divergence is to *S. cerevisiae*.


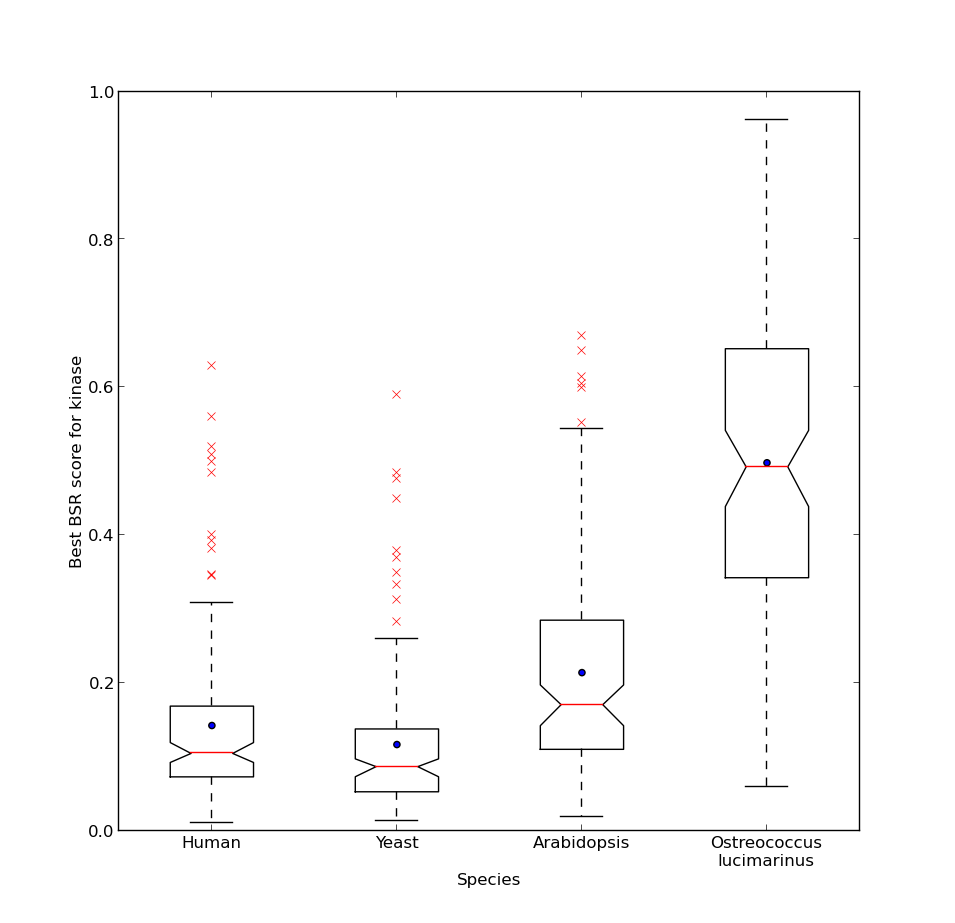


Table 1 Significance of the differences between species for the mean Blast Score Ratio (BSR) similarities of kinases. The p-values are calculated with a two-tailed Mann-Whitney U test comparing for each species the BSR similarities of kinases described previously. The comparison is between the vectors of BSR similarities, calculated from O. tauri protein kinases to the given species.

| Comparison | P-value |
| --- | --- |
| *S. cerevisiae - H. sapiens* | 0.01 |
| *A. thaliana* - *H. sapiens* | 2.67E-07 |
| *A. thaliana* - *S. cerevisiae* | 1.24E-12 |
