# Supplementary material for: The reduced kinome of Ostreococcus tauri: core eukaryotic signalling components in a tractable model species
Source: BMC Genomics. 2014 Aug 2;15:640. doi: 10.1186/1471-2164-15-640 (PMC4143559; doi:10.1186/1471-2164-15-640)
Supplement: Supplementary file 8 — Additional file 8: Figure S6: Discussion and phylogenies of further kinase families. Phylogenies and descriptions for the (A) TKL, (B) BUD32, (C) RIO, (D) STN, (E) SCYL (F) ABC1, and (G) Histidine Kinases families. (DOCX 692 KB) [file 12864_2014_6366_MOESM8_ESM.docx]

Further Kinase Families

# Eukaryotic Protein Kinases

## TKL kinases

The TKL tyrosine-like kinase family, unlike the TK family, is found throughout eukaryotes. In contrast to their structurally related TK family, TKL kinases contain mostly S/T kinases. While the TKL kinases in *O. tauri* are more similar to *A. thaliana* counterparts, there are no strongly conserved orthologs across eukaryotes. However, tyrosine phosphorylation is common in plants (de la Fuente van Bentem & Hirt, 2009), and it is likely that dual specificity kinases (DSP) replace the role of TKs in plants. We have observed tyrosine phosphorylation in *O. tauri*, and members of the DSP kinases families MAP2K, WEE1, DYRK, GSK3, TKL, BUD32, and CK2 kinases are all present in *O. tauri*.


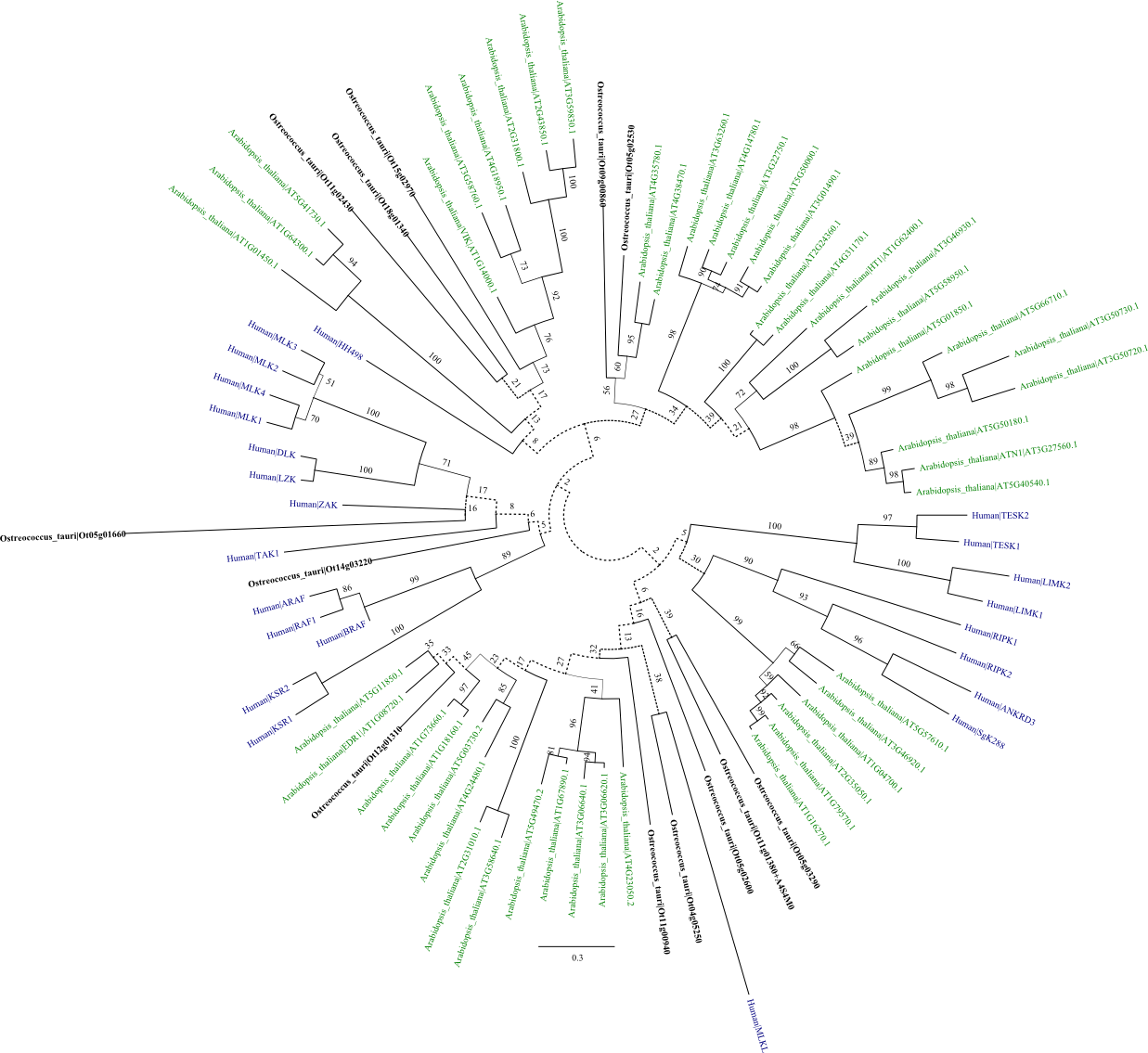
 Phylogeny of the TKL kinase group family. The tree shows *H. sapiens* (blue), *Arabidopsis thaliana* (green), and *Ostreococcus tauri* (bold) kinases. Confidence values from bootstrapping label the branches. The boldness of the branches is weighted from 100-40 confidence. Low confidence bootstrap values below 40% are shown with a broken line.

# Atypical kinases

## BUD32

The BUD32 protein kinase (Ot09g01420) in *O. tauri* is related to Bud site selection protein (BUD32) in yeast and human p53-related protein kinase (PRPK). BUD32 is found in almost all eukaryotes, in archaea, and some bacteria. Universally, it forms a complex or gene-fusion with kinase-associated endopeptidase 1 (Kae1) (Hecker *et al.,* 2009). Kae1 is also conserved in *O. tauri* (Ot13g00210). This complex is involved in telomere maintenance and transcriptional control in yeast (Hecker *et al.,* 2008; Kisseleva-Romanova *et al.,* 2006).


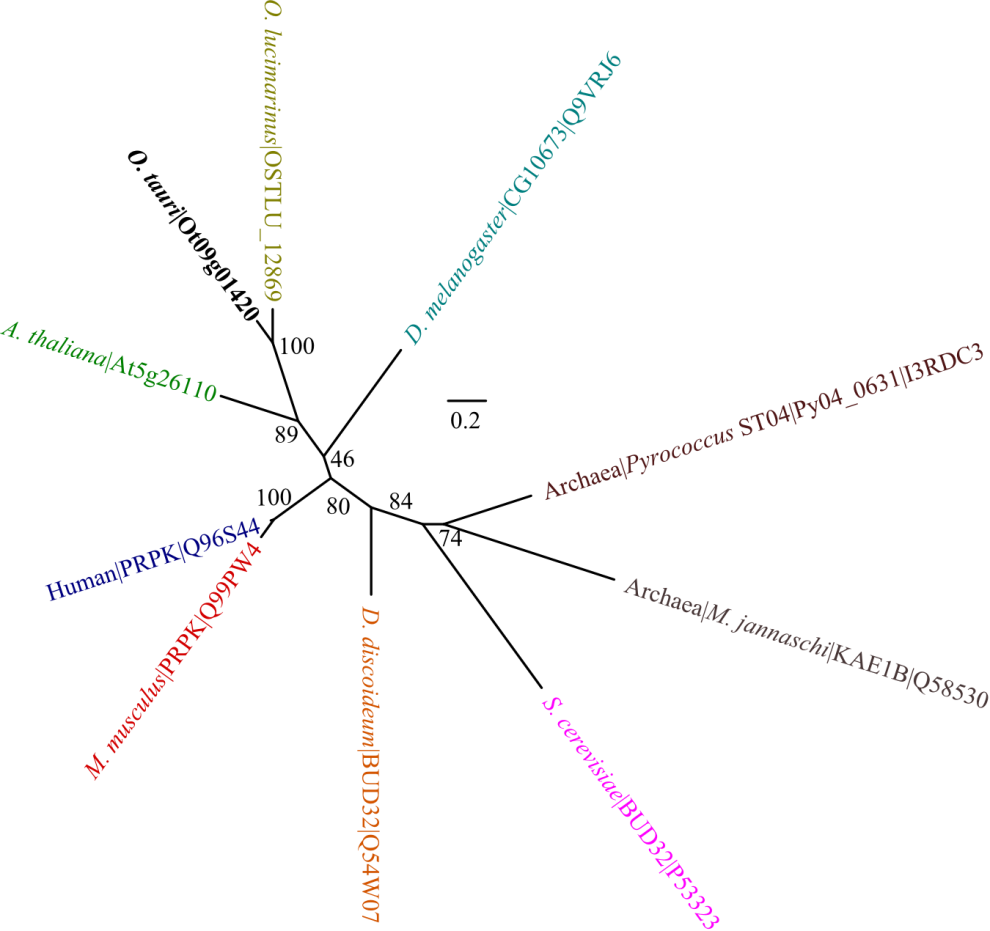


A phylogeny of the BUD32/PRPK protein kinases. Kinases are included from *O. tauri (bold)*, *O. lucimarinus (teal)*, *A. thaliana. (green)*, *H. sapiens* (blue), *S. cerevisiae* (magenta), *M. musculus* (red), *D. melanoganster* (cyan), slime mould (orange), and two Archaea protein sequences (brown). KAE1B is a fusion protein. The numbers attached to edges/branches are bootstrap values.

## RIO

The atypical Right Open reading frame (RIO) family of kinases were named after RIO1, which in yeast regulates checkpoints at two stages of the cell-cycle (Angermayr *et al.,* 2002). Both RIO1 and RIO2 are involved in ribosome biogenesis in yeast, and probably perform distinct roles as knock-out of either is lethal (LaRonde-LeBlanc & Wlodawer, 2005). Both RIO1 (Ot14g00390) and RIO2 (Ot06g01790) are present in *O. tauri*, supporting functional divergence of these two kinases.


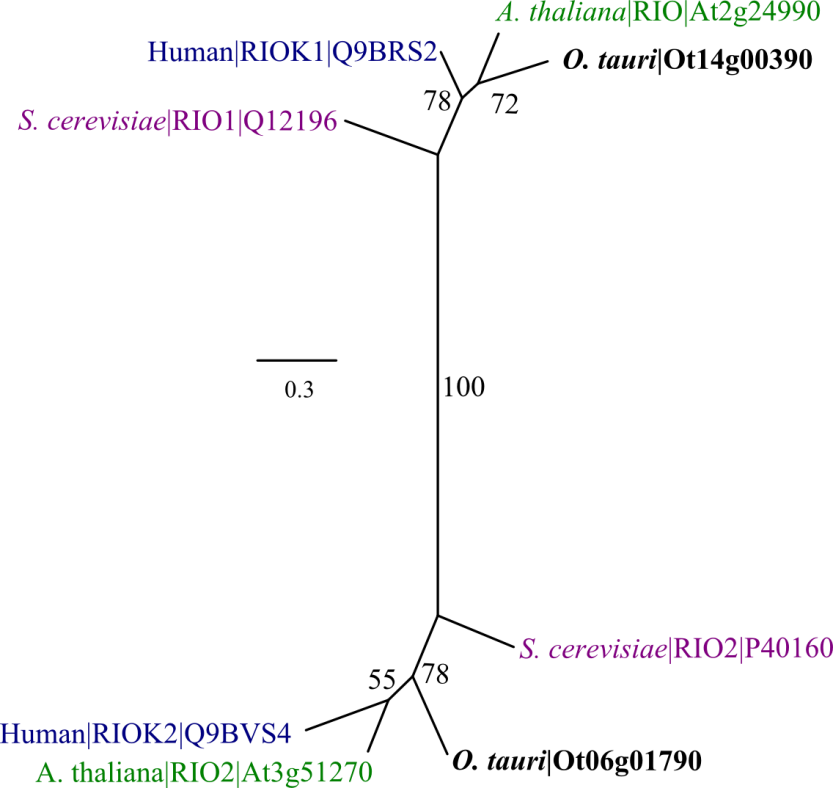


A phylogeny of the RIO protein kinases. Kinases are included from *O. tauri (bold)*, *H. sapiens* (blue), *S. cerevisiae* (magenta), and *A. thaliana. (green)*. The numbers attached to edges/branches are bootstrap values.

## STN

The State Transition (STN) family of kinases in *O. tauri* are related to STN7 and STN8 in *A. thaliana*. STN7 is required in higher-plants and algae for adaptation of the photosystem to different qualities of light (Bellafiore *et al.,* 2005; Depège *et al.,* 2003; Tikkanen *et al.,* 2010; Tikkanen & Aro, 2012). STN7 phosphorylates light harvesting proteins (LHC2), and STN8 phosphorylates core photosystem 2 (PS2) proteins (Bonardi *et al.,* 2005). The expanded STN family of proteins in *O. tauri* may be a function of the aquatic environment (Six *et al.,* 2009), and could provide an interesting insight into kinase control of photoadaptation.

## SCYL

Protein kinase-like SCY1-like (SCYL) proteins are highly conserved throughout eukaryotes, and may be pseudo-kinases (Scheeff *et al.,* 2009). Although their function is poorly understood, the high degree of conservation across eukaryotes and presence in the *O. tauri* minimal kinome potentially reflects essential conserved cellular roles that remain to be identified.

## ABC1

The ABC1 kinase family in algae is considerably larger than in other eukaryotes. Across the green lineage, the few ABC1 kinases that have been characterised are thought to be involved in stress responses (Yang, 2012), regulation of photosynthesis (Yang *et al.,* 2012b), and light sensing (Boyd *et al.,* 2011). We speculate that they may fulfil similar but expanded signalling requirements in algae.

## Histidine Kinases

Histidine Kinases (HKs) are an ancient family of proteins, pervasive across the tree of life, which typically mediate extracellular signalling through multi-component pathways, characterised by phosphorylation of a histidine residue. In *O. tauri* the HK sequences of components of a light-signalling pathway sit at structural midpoints in the phylogeny between cyanobacteria and land plants. The relative scarcity of components of the HK-mediated pathway in *O. tauri* makes it an attractive target for study. The *O. tauri* kinome contains two membrane-bound HK light receptors, LOV-HK and Rho-HK, and a single histidine-containing phosphotransfer (Hpt) factor. Hpts are typically cytoplasmic messengers down-stream of membrane-bound HKs. LOV-HK has already been shown to mediate light input into the circadian clock in *O. tauri* (Djouani-Tahri *et al.,* 2011). LOV-HK contains a LOV receptor domain, which is responsive to red light. Rho-HK contains a blue light responsive bacterial-like rhodopsin domain. A phylogeny of the HK domain of LOV-HK and Rho-HK demonstrates a close relationship to cytokinin-responsive HKs in *A. thaliana*. Both LOV-HK and Rho-HK contain C-terminal intracellular response domains, which typically interact with Hpt and facilitate the phosphorylation of a histidine in Hpt.


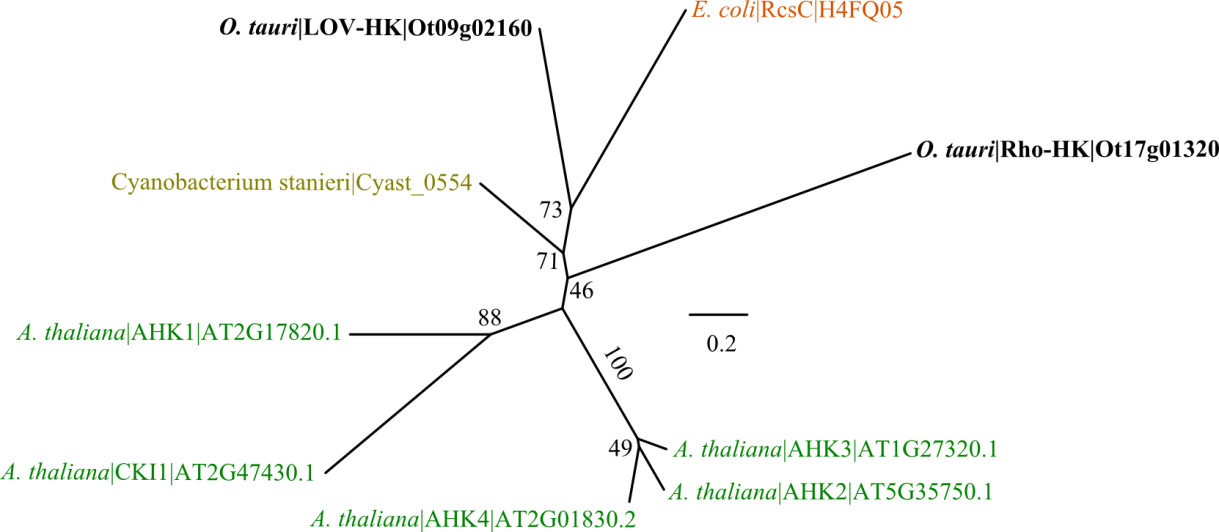


Supplemental Figure 1 A phylogeny of the HK protein kinases. Kinases are included from *O. tauri* (bold), and *A. thaliana.* (green), *Escherichia coli* (orange), and *Cyanobacterium stanieri* (yellow). The numbers attached to edges/branches are bootstrap values.

Histidine-containing phosphotransfer (Hpt) factors are related to histidine kinases, but contain large deletions within the kinase domain. *A. thaliana* contains six Hpt proteins, which are phosphorylated by cytokinin-responsive HKs. In *O. tauri*, there is only a single Hpt. This leads to the hypothesis, recently proposed by Pfeuty (2012), that both *O. tauri* light receptors share a single Hpt component. The high conservation of the HK domains within the HK receptor and Hpt phylogenies of *O. tauri* and the HK domains of cytokinin signalling in *A. thaliana*, likely reflect that *O. tauri* components are prototypical for this signalling pathway.


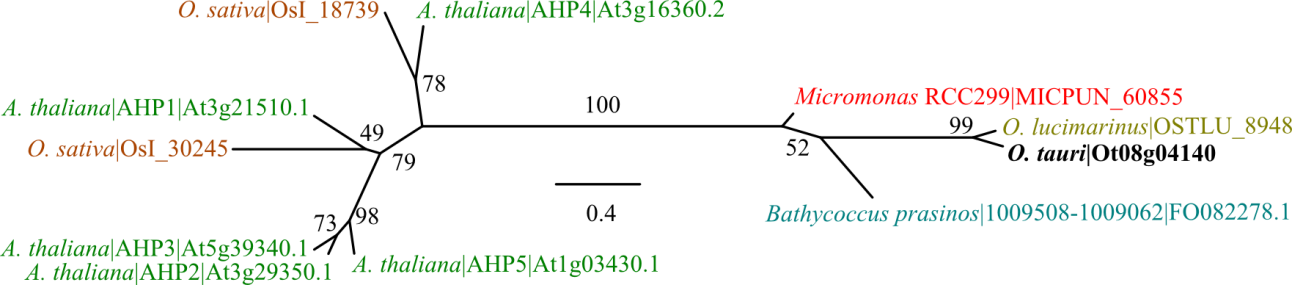


Supplemental Figure 2 A phylogeny of the histidine-containing phosphotransfer (HPt) protein. Proteins are included from *O. tauri (bold)*, *O. lucimarinus (teal)*, and *A. thaliana. (green)*, *O. sativa* (orange), *Micromonas* RCC299 (red), and *Bathycoccus* *prasinos* (blue). The *Bathycoccus* identifier refers to the genomic coordinates on the referenced scaffold, as there was no published gene model for this protein. The numbers attached to edges/branches are bootstrap values.

In *A. thaliana* 18 response regulators (ARR) translate Hpt activation into transcriptional changes. In *O. tauri,* there is only one candidate ARR protein (Ot16g01320), which contains a response regulator and Myb transcription factor domain). An APRR (TOC1) is also present in *O. tauri* and forms a central component of the transcriptional circadian clock circuitry (Troein *et al.,* 2011; Corellou *et al.,* 2009). TOC1 is therefore an obvious candidate for an input of light into the transcriptional clock, mediated by histidine kinase signalling. Similar mechanistically to HKs: Pyruvate Dehydrogenase Kinase (PDK) phosphorylates and deactivates pyruvate dehydrogenase across eukaryotes (Thelen *et al.,* 2000; Korotchkina & Patel, 2001), and works reversibly with pyruvate dehydrogenase phosphatase (PDPC) (Holness & Sugden, 2003), also known as Protein Phosphatase 2C (PP2C), to regulate glucose homeostasis. Interestingly, while PDK phosphorylates S/T residues, mechanistically it works very similar to Histidine Kinases (Mooney *et al.,* 2000).

# Other Kinases

In addition to CMGC kinases, the protein kinase families BUB1, Haspin, and Polo-like kinases (PLK) are also involved in cell-cycle control. The importance of protein kinases to regulate the cell cycle is demonstrated in the quantity and diversity of families of cell cycle protein kinases in *O. tauri*. Significant progress has already been made on the study of components and properties of the *O. tauri* cell cycle (Farinas *et al.,* 2006; Corellou *et al.,* 2005; Robbens *et al.,* 2005).

## BUB

The BUB1-like protein kinase (Ot12g00570) is named after the Budding Uninhibited by Benzimidazoles 1 (BUB1) protein kinase in yeast, and has been identified across the green lineage (Karpov *et al.,* 2010). BUB1 is involved in the spindle assembly checkpoint (SAC) and DNA damage response (DDR) (Yang *et al.,* 2012a). Two phosphatidyl inositol 3' kinase-related kinases (PIKK) kinases are also associated with the mitotic checkpoint control and DDR (Abraham, 2001). BUB1 has been suggested to be an ATM and ATR target (Matsuoka *et al.,* 2007).

## Haspin

Haspin is another *O. taur*i kinase that is associated with spindle formation during mitosis (Dai *et al.,* 2005). It is conserved in many eukaryotes, including plants (At1g09450), human, yeast (ALK1), and *O. tauri* (Ot05g03870).

## PLK

Polo-like kinases (PLK) are involved at several stages throughout the cell cycle (Glover *et al.,* 1998). *O. tauri,* like most eukaryotes, only contains one copy of PLK (Ot04g00800). PLK is activated by Aurora (AUR) kinase, which thereby controls entry into mitosis (Seki *et al.,* 2008). *O. tauri* contains a single ortholog of AUR (Ot10g01060), and an AUR-like protein kinase (CALK) (Ot13g01960). The latter is conserved throughout algae, and has been show to regulate flagella length in *Chlamydomonas* (Luo *et al.,* 2011; Pan *et al.,* 2004). Mammalian AurA conducts a similar role in cilium disassembly (Pugacheva *et al.,* 2007). The presence of CALK in algae devoid of flagella, like *O. tauri*, indicates an additional role for CALK.

# References

Abraham RT (2001) Cell cycle checkpoint signaling through the ATM and ATR kinases. *Genes Dev.* **15:** 2177–2196

Angermayr M, Roidl A & Bandlow W (2002) Yeast Rio1p is the founding member of a novel subfamily of protein serine kinases involved in the control of cell cycle progression. *Mol. Microbiol.* **44:** 309–324

Bellafiore S, Barneche F, Peltier G & Rochaix J-D (2005) State transitions and light adaptation require chloroplast thylakoid protein kinase STN7. *Nature* **433:** 892–895

Bonardi V, Pesaresi P, Becker T, Schleiff E, Wagner R, Pfannschmidt T, Jahns P & Leister D (2005) Photosystem II core phosphorylation and photosynthetic acclimation require two different protein kinases. *Nature* **437:** 1179–1182

Boyd JS, Mittelmeier TM, Lamb MR & Dieckmann CL (2011) Thioredoxin-family protein EYE2 and Ser/Thr kinase EYE3 play interdependent roles in eyespot assembly. *Mol. Biol. Cell* **22:** 1421–1429

Corellou F, Camasses A, Ligat L, Peaucellier G & Bouget F-Y (2005) Atypical regulation of a green lineage-specific B-type cyclin-dependent kinase. *Plant Physiol.* **138:** 1627–1636

Corellou F, Schwartz C, Motta J-P, Djouani-Tahri EB, Sanchez F & Bouget F-Y (2009) Clocks in the green lineage: comparative functional analysis of the circadian architecture of the picoeukaryote ostreococcus. *Plant Cell* **21:** 3436–3449

Dai J, Sultan S, Taylor SS & Higgins JMG (2005) The kinase haspin is required for mitotic histone H3 Thr 3 phosphorylation and normal metaphase chromosome alignment. *Genes Dev.* **19:** 472–488

Depège N, Bellafiore S & Rochaix J-D (2003) Role of Chloroplast Protein Kinase Stt7 in LHCII Phosphorylation and State Transition in Chlamydomonas. *Science* **299:** 1572–1575

Djouani-Tahri E-B, Christie JM, Sanchez-Ferandin S, Sanchez F, Bouget F-Y & Corellou F (2011) A eukaryotic LOV-histidine kinase with circadian clock function in the picoalga Ostreococcus. *Plant J.* **65:** 578–588

Farinas B, Mary C, de O Manes C-L, Bhaud Y, Peaucellier G & Moreau H (2006) Natural synchronisation for the study of cell division in the green unicellular alga Ostreococcus tauri. *Plant Mol. Biol.* **60:** 277–292

De la Fuente van Bentem S & Hirt H (2009) Protein tyrosine phosphorylation in plants: More abundant than expected? *Trends Plant Sci.* **14:** 71–76

Glover DM, Hagan IM & Tavares ÁAM (1998) Polo-like kinases: a team that plays throughout mitosis. *Genes Dev.* **12:** 3777–3787

Hecker A, Graille M, Madec E, Gadelle D, Le Cam E, van Tilbergh H & Forterre P (2009) The universal Kae1 protein and the associated Bud32 kinase (PRPK), a mysterious protein couple probably essential for genome maintenance in Archaea and Eukarya. *Biochem. Soc. Trans.* **37:** 29

Hecker A, Lopreiato R, Graille M, Collinet B, Forterre P, Libri D & van Tilbeurgh H (2008) Structure of the archaeal Kae1/Bud32 fusion protein MJ1130: a model for the eukaryotic EKC/KEOPS subcomplex. *EMBO J.* **27:** 2340–2351

Holness MJ & Sugden MC (2003) Regulation of pyruvate dehydrogenase complex activity by reversible phosphorylation. *Biochem. Soc. Trans.* **31:** 1143–1151

Karpov PA, Raevskiĭ AV & Blium IB (2010) [Bioinformatic search for plant homologs of protein kinase BUB1--the keypoint of mitotic spindle assembly]. *T͡Sitologii͡a Genet.* **44:** 57–69

Kisseleva-Romanova E, Lopreiato R, Baudin-Baillieu A, Rousselle J-C, Ilan L, Hofmann K, Namane A, Mann C & Libri D (2006) Yeast homolog of a cancer-testis antigen defines a new transcription complex. *EMBO J.* **25:** 3576–3585

Korotchkina LG & Patel MS (2001) Site specificity of four pyruvate dehydrogenase kinase isoenzymes toward the three phosphorylation sites of human pyruvate dehydrogenase. *J. Biol. Chem.* **276:** 37223–37229

LaRonde-LeBlanc N & Wlodawer A (2005) A Family Portrait of the RIO Kinases. *J. Biol. Chem.* **280:** 37297–37300

Luo M, Cao M, Kan Y, Li G, Snell W & Pan J (2011) The phosphorylation state of an aurora-like kinase marks the length of growing flagella in Chlamydomonas. *Curr. Biol. CB* **21:** 586–591

Matsuoka S, Ballif BA, Smogorzewska A, McDonald ER, Hurov KE, Luo J, Bakalarski CE, Zhao Z, Solimini N, Lerenthal Y, Shiloh Y, Gygi SP & Elledge SJ (2007) ATM and ATR Substrate Analysis Reveals Extensive Protein Networks Responsive to DNA Damage. *Science* **316:** 1160–1166

Mooney BP, David NR, Thelen JJ, Miernyk JA & Randall DD (2000) Histidine modifying agents abolish pyruvate dehydrogenase kinase activity. *Biochem. Biophys. Res. Commun.* **267:** 500–503

Pan J, Wang Q & Snell WJ (2004) An aurora kinase is essential for flagellar disassembly in Chlamydomonas. *Dev. Cell* **6:** 445–451

Pfeuty B, Thommen Q, Corellou F, Djouani-Tahri EB, Bouget F-Y & Lefranc M (2012) Circadian clocks in changing weather and seasons: lessons from the picoalga Ostreococcus tauri. *BioEssays News Rev. Mol. Cell. Dev. Biol.* **34:** 781–790

Pugacheva EN, Jablonski SA, Hartman TR, Henske EP & Golemis EA (2007) HEF1-dependent Aurora A activation induces disassembly of the primary cilium. *Cell* **129:** 1351–1363

Robbens S, Khadaroo B, Camasses A, Derelle E, Ferraz C, Inzé D, Van de Peer Y & Moreau H (2005) Genome-wide analysis of core cell cycle genes in the unicellular green alga Ostreococcus tauri. *Mol. Biol. Evol.* **22:** 589–597

Scheeff ED, Eswaran J, Bunkoczi G, Knapp S & Manning G (2009) Structure of the pseudokinase VRK3 reveals a degraded catalytic site, a highly conserved kinase fold, and a putative regulatory binding site. *Struct. Lond. Engl. 1993* **17:** 128–138

Seki A, Coppinger JA, Jang C-Y, Yates JR & Fang G (2008) Bora and the kinase Aurora a cooperatively activate the kinase Plk1 and control mitotic entry. *Science* **320:** 1655–1658

Six C, Sherrard R, Lionard M, Roy S & Campbell DA (2009) Photosystem II and Pigment Dynamics among Ecotypes of the Green Alga Ostreococcus. *Plant Physiol.* **151:** 379–390

Thelen JJ, Miernyk JA & Randall DD (2000) Pyruvate dehydrogenase kinase from Arabidopsis thaliana: a protein histidine kinase that phosphorylates serine residues. *Biochem. J.* **349:** 195–201

Tikkanen M & Aro E-M (2012) Thylakoid protein phosphorylation in dynamic regulation of photosystem II in higher plants. *Biochim. Biophys. Acta* **1817:** 232–238

Tikkanen M, Grieco M, Kangasjärvi S & Aro E-M (2010) Thylakoid Protein Phosphorylation in Higher Plant Chloroplasts Optimizes Electron Transfer under Fluctuating Light. *Plant Physiol.* **152:** 723–735

Troein C, Corellou F, Dixon LE, van Ooijen G, O’Neill JS, Bouget F-Y & Millar AJ (2011) Multiple light inputs to a simple clock circuit allow complex biological rhythms. *Plant J. Cell Mol. Biol.* **66:** 375–385

Yang C, Wang H, Xu Y, Brinkman KL, Ishiyama H, Wong STC & Xu B (2012a) The kinetochore protein Bub1 participates in the DNA damage response. *DNA Repair* **11:** 185–191

Yang S (2012) AtSIA1, an ABC1-like kinase, regulates salt response in Arabidopsis. *Biologia (Bratisl.)* **67:** 1107–1111

Yang S, Zeng X, Li T, Liu M, Zhang S, Gao S, Wang Y, Peng C, Li L & Yang C (2012b) AtACDO1, an ABC1-like kinase gene, is involved in chlorophyll degradation and the response to photooxidative stress in Arabidopsis. *J. Exp. Bot.* **63:** 3959–3973
